# Human In Vivo Metabolism and Elimination Behavior of Micro-Dosed Selective Androgen Receptor Modulator RAD140 for Doping Control Purposes

**DOI:** 10.3390/metabo12070666

**Published:** 2022-07-20

**Authors:** Felicitas Wagener, Luisa Euler, Christian Görgens, Sven Guddat, Mario Thevis

**Affiliations:** 1 Institute of Biochemistry/Center for Preventive Doping Research, German Sports University Cologne, Am Sportpark Müngersdorf 6, 50933 Cologne, Germany; f.wagener@biochem.dshs-koeln.de (F.W.); l.euler@biochem.dshs-koeln.de (L.E.); c.goergens@biochem.dshs-koeln.de (C.G.); s.guddat@biochem.dshs-koeln.de (S.G.); 2 European Monitoring Center for Emerging Doping Agents (EuMoCEDA), 50933 Cologne, Germany

**Keywords:** selective androgen receptor modulators (SARMs), doping, RAD140, metabolism, elimination profile

## Abstract

RAD140 is a selective androgen receptor modulator which has been abused in sporting competitions. Its use is prohibited by the World Anti-Doping Agency (WADA) for athletes at all times. In addition to its illicit use, adverse analytical findings of RAD140 in doping control samples might result from other scenarios, e.g., the ingestion of contaminated dietary supplements. The differentiation between samples resulting from such contamination scenarios and intentional doping presents a considerable challenge, as little is known about the metabolism and elimination behavior of RAD140 in humans. In this study, six micro-dose excretion studies with five adult male volunteers each were conducted, and urine samples were analyzed by means of LC-HRMS/MS. Multiple metabolites, firstly detected in human urine, are described in this study. The sample preparation included an enzymatic hydrolysis step, which facilitated the estimation of RAD140 concentrations in urine. The elimination profiles and detection times for six metabolites as well as the intact drug are presented. The method was extensively characterized and deemed fit-for-purpose. The metabolite ratios were investigated for their predictive power in estimating the dose of RAD140 intake. The presented data will aid in better case result management in future doping cases involving RAD140.

## 1. Introduction

Selective androgen receptor modulators (SARMs) are a class of novel androgenic drugs that are being developed for multiple applications such as androgen replacement therapy, the treatment of cancer, and muscle wasting [1,2]. SARMs are mostly non-steroidal and vary widely in their structure. What all SARMs have in common is their agonistic effect on the androgen receptor (AR), while being tissue-selective. SARMs are designed to be AR agonists in tissues such as muscle and bone while showing reduced or even antagonistic activity in tissues such as prostate, seminal vesicles, and skin [3].

As SARMs promote muscle growth and strength, increasing interest was noted concerning individuals looking for performance-enhancing substances, especially in strength-based sports, such as weightlifting or bodybuilding. The World Anti-Doping Agency (WADA) has included SARMs in the Prohibited List since 2008, and the number of SARMs explicitly mentioned has substantially increased over the last years [4]. On the latest Prohibited List, andarine, enobosarm (ostarine), LGD-4033 (ligandrol), and RAD140 are featured [5].

**Figure 1:**
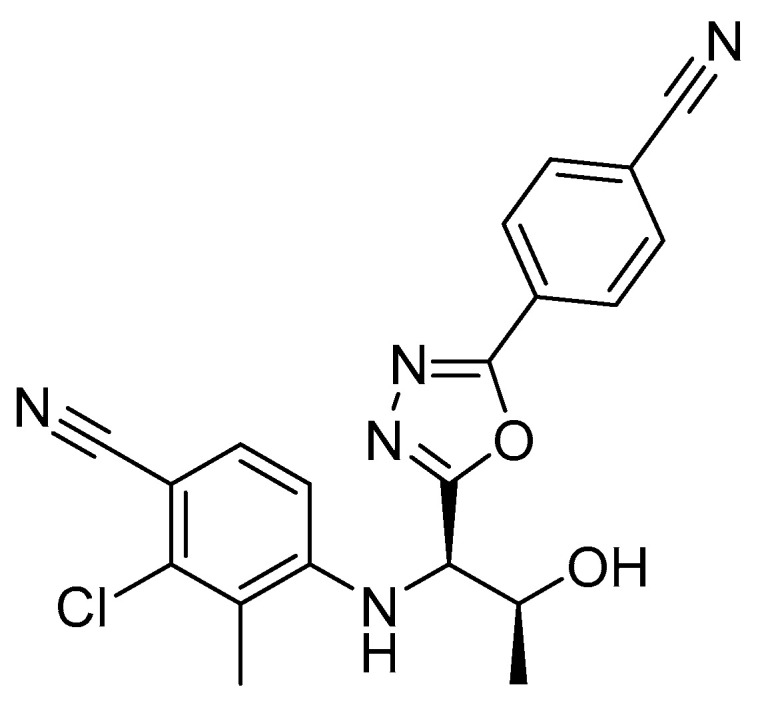
Chemical structure of RAD140 [6].

RAD140 is a SARM that was first described by Miller et al. in 2010 (Figure 1) [6]. Preclinical data in rats and monkeys showed anabolic effects on muscle while showing a reduced effect on prostate growth when compared to testosterone. Additionally, RAD140 showed good pharmacokinetic properties [6]. A phase I study on the efficacy of RAD140 in the treatment of ER+/HER2- metastatic breast cancer revealed an acceptable safety profile and preliminary evidence of antitumor activity. Therein, doses between 50 mg and 150 mg were administered to breast cancer patients, and a half-life of 45 h was observed [7]. As RAD140 has so far not gained clinical approval, detailed information on the pharmacokinetics and drug–drug interactions of RAD140 is scarce. Although SARMs show reduced side effects in comparison to anabolic androgenic steroids due to their tissue-selective mechanism of action, case reports have been published which link the intake of RAD140 to myocarditis and liver injury [8,9].

The mass spectrometric behavior of RAD140 in regard to sports drug testing was characterized by Thevis et al. in 2013 [10]. A dissociation pathway under positive electrospray ionization (ESI) conditions was proposed with the main product ions at *m*/*z* 223, 205, and 172 resulting from the fragmentation of the protonated molecule [M + H]^+^ at *m*/*z* 394. Additionally, RAD140 is detectable in negative ESI mode after the rapid elimination of acetaldehyde to yield *m*/*z* 348. The main product ions in the negative mode are *m*/*z* 321, 175, 165, and 127 [11].

On the metabolism of RAD140, a limited amount of data is available so far. So et al. investigated the in vitro and in vivo metabolism of RAD140 in horses [12]. Multiple metabolites were discovered in vitro and confirmed in in vivo studies. To our knowledge, the only data on human metabolism were presented in the contribution of Sobolevsky et al., in which low amounts of mono- and bis-hydroxylated metabolites were detected in vitro [11]. In vivo, two closely eluting isomers of mono-hydroxylated RAD140 were detected with product ions *m*/*z* 193, 191, 180, and 144, resulting from the precursor *m*/*z* 364 after the elimination of acetaldehyde.

In addition to deliberate doping with SARMs, a problem that has garnered increasing attention is athletes’ exposome, and especially the risk contaminated supplements pose to athletes [13,14]. As dietary supplements, in contrast to pharmaceuticals, are not extensively monitored, they may contain undeclared prohibited ingredients [15]. This can lead to adverse analytical findings (AAFs) and thus the suspension of athletes from competitions. Under WADA regulations, athletes are solely responsible for the substances in their bodies and have to prove “no fault or negligence” or “no significant fault or negligence” to suspend or reduce their sanction (WADAs principle of “Strict Liability”) [16]. To be able to differentiate between deliberate doping and the intake of small amounts of prohibited substances via contaminated supplements or other scenarios of drug exposure, information about metabolic fate and excretion behavior is indispensable. For the SARMs ostarine and LGD-4033, controlled excretion studies have been conducted previously [17,18]. For LGD-4033, the time of intake can be estimated by comparing metabolite ratios that change over time after the ingestion of the drug. These kinds of studies may lead to better case result management by doping control authorities and assist in protecting the clean athlete. The aim of this study is to advance the knowledge regarding the metabolism of the SARM RAD140 and to establish a liquid chromatography–high resolution tandem mass spectrometry (LC-HRMS/MS) method to detect RAD140 and its metabolites in human urine samples.

## 2. Results and Discussion

### 2.1. Determination of Urinary Metabolites of RAD140

To gain insight into the metabolism of RAD140, both micro-dose excretion urines and one routine doping control sample (RDS) were available for method development. The athlete from whom the RDS was collected declared consent for research purposes. The RDS resulted in an AAF in 2020 and contained an estimated amount of 4 μg/mL RAD140. Since this is a much higher RAD140 concentration than in the samples obtained after micro-dosing, these samples are not expected to contain all the metabolites found in the RSD.

**Figure 2:**
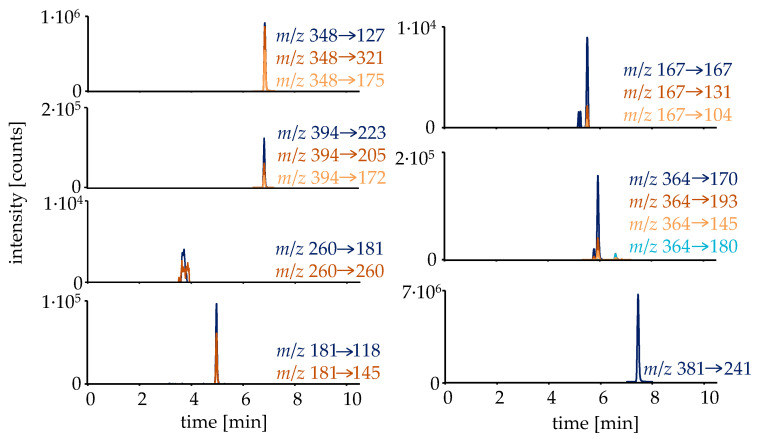
Extracted ion chromatograms of the detected analytes in a urine sample collected 14.5 h after intake of a single dose of 50 μg of RAD140. The sample was prepared via hydrolysis and LLE.

The first step in investigating the metabolism and elimination behavior of RAD140 was to curate a list of possible metabolites. For this, additionally to already described metabolites in horses and humans, metabolites were formed in silico by employing the online biotransformer tool [19]. All detected metabolites are listed in Table 1. The detected metabolites were analyzed in targeted HRMS/MS mode with normalized collision energies (NCEs) optimized for each analyte. To facilitate an estimation of the RAD140 concentration in the micro-dose study samples, a sample preparation including a hydrolysis step followed by liquid–liquid extraction (LLE) was employed, deconjugating the main metabolite M4 into the parent compound, for which a reference standard was available. The extracted ion chromatograms of the metabolites that were detected in the micro-dose study samples are shown in Figure 2.

For the detection of the Phase I and Phase II metabolites in the RDS, the sample was measured both by direct injection and after work-up with hydrolysis and LLE. The chromatograms of the RDS are shown in the Supplementary Materials Figure S13. The HRMS/MS spectra of all detected metabolites are shown in the Supplementary Material Figures S1–S12. The proposed structures and metabolic pathways are shown in Figure 3.

Metabolites M1, M3, M4, and M5 were previously described in horses by So et al. [12]. For M5, a reference standard was available; therefore, the identity of M5 could be verified as 4-amino-2-chloro-3-methylbenzonitrile. Of the monohydroxylated metabolites M6a, M6b, and M6c, only two isomers were reported by Sobolevsky et al. [11]. The hydroxylated and glucuronidated metabolites M2a and M2b and the sulfated metabolite M7 are herein described for the first time as metabolites of RAD140.

### 2.2. Method Characterization for the Detection of RAD140

The developed method was characterized extensively based on the WADA criteria with added parameters where deemed necessary. All characterization parameters are described in Section 3.5: Method Characterization. The results of the method characterization can be found in Table 2. The limit of detection (LOD) and the limit of identification (LOI) were determined at 9 pg/mL and 120 pg/mL, respectively. The method was confirmed to be selective for the detection of RAD140. Carryover, robustness, and the standard deviation of the retention times were all found to be acceptable. The parameters recovery and matrix effects showed some variability, but as the imprecision lay within the acceptance criteria, these variations were well compensated by the ISTD. As the stability decreased over time, a freshly prepared calibration curve was prepared with every batch of samples to compensate for the reduced benchtop stability. The method was shown to be highly linear, with values of R^2^ > 0.99. Therefore, this method was determined to be suitable for the detection of RAD140. The concentration of RAD140 was estimated in the samples using the calibration curves.

### 2.3. Elimination Data of Micro-Dose Studies of RAD140

**Figure 4:**
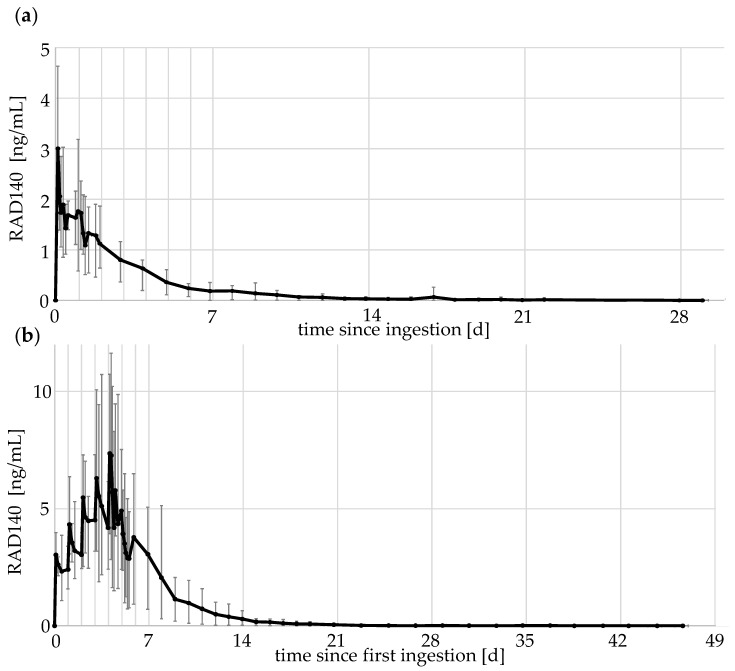
Elimination profile of RAD140 (**a**) after the intake of a single dose of 50 μg of RAD140 (**b**) after the intake of five doses of 50 μg of RAD140 over 5 days. The black line indicates the average values of the five volunteers; minimum and maximum values are shown as error bars.

For the micro-dose excretion studies, elimination profiles of the different doses were compiled. RAD140 can be ionized in both positive and negative mode using ESI, whereby the compound is detected as protonated molecule [M + H]^+^ or as in-source fragment [M-C_2_H_6_O]^−^, respectively. The detection of both precursor ions of RAD140 has been described previously [11,12]. Both polarities were used to measure the parent compound, but as the negative ESI mode was about 10-fold more sensitive than the positive mode, the results in the negative mode were used to estimate concentrations of RAD140. The concentration of RAD140 was estimated using a calibration curve prepared the same day. The elimination profiles of the 50 μg single- and multi-dose study are shown in Figure 4. The elimination profiles of the other doses can be found in the Supplementary Materials Figures S14–S17.

For the elimination profiles, the data of all five volunteers were aggregated, with the average values shown in black and maximum and minimum values shown as error bars. The elimination shows a rapid increase in RAD140 concentration in urine after the ingestion of micro-doses. The concentration reaches maximum values 2–6 h after ingestion. Afterwards, concentrations decrease slowly with detection times of up to 29 days for a single 50 μg application. When taken 24 h apart, RAD140 shows accumulation over the five application days. For the 50 μg dose, the average value measured 2 h after the fifth application is 2.4-fold higher than the average value determined 2 h after the first application (3.0 ng/mL vs. 7.3 ng/mL).

Maximum concentrations reached 0.06 ng/mL and 0.17 ng/mL after the single- and multi-dose application of 1 μg of RAD140, 0.9 ng/mL and 1.2 ng/mL after the single- and multi-dose application of 10 μg of RAD140, and 4.6 ng/mL and 11.6 ng/mL after the single- and multi-dose application of 50 μg of RAD140, respectively. The detailed detection times of all detected metabolites are summarized in Table 3.

As the parent compound RAD140 is detected the longest after ingestion, it is the preferable analyte for doping control purposes, as it offers the highest retrospectivity. However, as M6b offers the longest detection time of all observed metabolites, it may also prove to be a valuable additional target in routine doping control testing procedures.

### 2.4. Metabolite Ratios of RAD140

**Figure 5:**
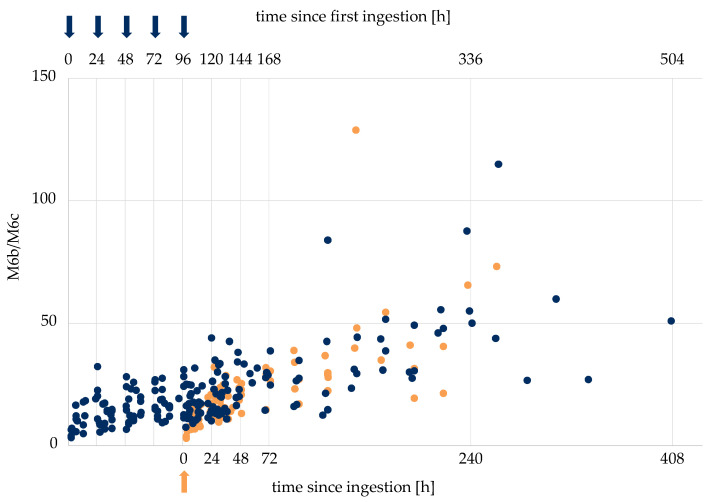
Metabolite ratios M6b/M6c of single application of RAD140 (orange) and multi-dose application of RAD140 (blue). Arrows indicate the intake of 50 μg of RAD140.

It has been shown in previous studies that metabolite ratios of excretion studies may assist case result management in the context of doping controls [18]. Therein, the ratio of LGD-4033 and its epimer were used to estimate the time and dose of SARM intake, to support the differentiation between samples taken a short time after the ingestion of a small dose of LGD-4033 and later samples taken after the dosage of amounts compatible with the intent to dope. To investigate if the same approach is possible for RAD140, metabolite ratios of different metabolites of RAD140 were generated using the measured peak areas and investigated. As most metabolites are not detectable in the lower-dosage application studies, the data from the 50 μg single- and multi-dose studies were used (*n* = 5 + 5). The ratios of the single- and multi-dose studies were combined by lining up the values of the single application with the last ingestion of the multi-dose application. Multiple ratios show a correlation to the time of intake. The ratios M6b/M6c, M6a/M6c, M6a/M6b, M6b/M3, M6a/M3, M6a/RAD140, and M6b/RAD140 all showed higher values at later time points compared to analytical data obtained shortly after the ingestion of RAD140. Exemplarily, the ratios of M6b/M6c are shown in Figure 5, and additional ratios are shown in the Supplementary Material Figures S18–S23. In Table 4, tentative “threshold ratios” for the different metabolite combinations are shown. Here, all ratios that are below or above the listed value correspond to samples taken in the indicated time frame (<48 h, <72 h, or >96 h) after the last intake of 50 μg of RAD140.

These data suggest that lower values of the aforementioned threshold ratios may indicate a shorter period between the intake of RAD140 and sample collection. However, as the total metabolite concentration is low and the ratios vary between volunteers, this method does not allow for a definitive statement about the time of intake and dose ingested. Further controlled administration studies with larger amounts of RAD140 are necessary to corroborate the threshold ratios shown in Table 4. Nonetheless, this first data set regarding the elimination behavior of different metabolites of RAD140, in combination with the RAD140 concentration, may help in reviewing statements by athletes that RAD140 was ingested deliberately.

## 3. Materials and Methods

### 3.1. Chemicals and Reagents

Standard material of RAD140 and 4-amino-2-chloro-3-methylbenzonitrile was obtained from Selleckchem (Houston, TX, USA) and USBiological (Salem, MA, USA), respectively. Ultrapure water was generated in-house with a BarnsteadGenPure xCAD Plus from Thermo Scientific (Bremen, Germany). As the ISTD, a 50 ng/mL solution of arylpropionamide-derived SARM S-24 in acetonitrile (ACN) was used. S-24 was synthesized in-house following a previously published procedure [20]. ACN was obtained from VWR chemicals (Radnor, PA, USA). Ethanol (EtOH) was purchased from Merck (Darmstadt, Germany). *Tert*-butyl methyl ether (*t*BME) was obtained from PanReac AppliChem (Darmstadt, Germany). β-Glucuronidase (Escherichia coli) was obtained from Roche Diagnostics (Mannheim, Germany). Formic acid (FA) was purchased from Sigma Aldrich (St. Louis, MO, USA). Phosphate buffer was prepared by combining 17.3 g of disodium hydrogen phosphate (VWR, Radnor, PA, USA) with 8.8 g of sodium dihydrogen phosphate monohydrate (Merck, Darmstadt, Germany) and 230 mL of ultrapure water. Ammonium acetate buffer (5 mM, pH 3.5) was prepared with ammonium acetate (NH_4_Ac) provided by Merck (Darmstadt, Germany) and acetic acid (AcOH) from Sigma Aldrich (St. Louis, MO, USA). Carbonate buffer was prepared by mixing 30 g of potassium hydrogencarbonate with 30 g of potassium carbonate (both VWR, Radnor, PA, USA) and 240 mL of ultrapure water. For all volume measurements, Research plus pipettes from Eppendorf (Hamburg, Germany) with a maximum deviation of ± 1% were used.

### 3.2. Sample Collection

Five healthy male volunteers each were recruited for the 1, 10, and 50 μg single- and multi-dose studies with RAD140 (six studies with thirty study participants in total). Written informed consent was obtained prior to the application. A 1 mg/mL stock solution of RAD140 was prepared in EtOH and spiked into 120 mL of drinking yoghurt at the required dose. Each volunteer collected one blank urine sample before ingesting the spiked yoghurt. After the single-dose application, a sample of every instance of urine excreted was collected for the first 48 h, roughly every 2 hours during the day, followed by one urine sample per day, until no analyte attributed to the RAD140 administration was detected in urine. For the multiple-application studies, five doses of RAD140 were ingested on five consecutive days. During the time of applications, four urine samples a day were collected. After the last ingestion, every urine sample was collected for 48 h, roughly every 2 hours during the day, followed by one urine sample per day, until the target analytes were not detected anymore in urine. The samples were stored at 4 °C for a maximum of 7 days, after which, they were frozen at −20 °C for long-term storage. All values were corrected for specific gravity (SG) using the equation below. SG was measured with an ORF 1PM digital refractometer from Kern optics (Balingen, Germany).

$${\mathrm{Concentration}}_{\mathrm{adjusted}}=\frac{\left(1.020-1\right)}{\left({\mathrm{SG}}_{\mathrm{sample}}-1\right)}\cdot {\mathrm{Concentration}}_{\mathrm{measured}}$$

### 3.3. Sample Preparation

Prior to analysis, the samples were thawed at room temperature. To 2 mL of the homogenized sample, 10 μL of ISTD, 1 mL of phosphate buffer (pH 7), and 50 μL of β-glucuronidase was added. The sample was vortexed and hydrolyzed at 50 °C for 60 min. After hydrolysis, 0.75 mL of carbonate buffer and 5 mL of *t*BME were added. The samples were shaken for 5 min before being centrifuged at 1800× *g* for 5 min in a Multifuge 1 S-R from Heraeus (Hanau, Germany). The organic phase was removed and evaporated to dryness. The sample was reconstituted in 100 μL of ACN/5 mM NH_4_Ac buffer 10/90 (*v*:*v*) before analysis via LC-HRMS/MS.

### 3.4. Liquid Chromatography–High-Resolution Tandem Mass Spectrometry

The analysis was conducted on an Exploris 480 Orbitrap mass spectrometer coupled to a Vanquish UHPLC system, both from Thermo Scientific (Bremen, Germany). A Poroshell 120 EC-C18 (3.0 × 50 mm, 2.7 μm) was used as the analytical column with a UHPLC Guard column of the same material (3.0 × 5 mm, 2.7 μm), both from Agilent (Santa Clara, CA, USA). As the mobile phase, 0.1% FA was used as eluent A and ACN was used as eluent B. A linear gradient was employed for the separation, starting at 20% B going to 50% B in 4.5 min, increasing to 100% B in another 2.5 min and holding there for 1 min, followed by 2.5 min re-equilibration at 20% B. A flow rate of 0.3 mL/min was applied over the whole gradient. The total run time of the gradient amounted to 10.5 min. For each measurement, 5 μL of sample was injected onto the LC column. The MS was operated in both the positive and negative ionization mode using a heated ESI source. The ionization was conducted with 3500 V in the positive mode and −3000 V in the negative mode. The vaporizer temperature was set to 300 °C. The full-scan experiments were conducted with a resolution of 30,000 FWHM and a scan range of *m*/*z* 80–800. HRMS/MS experiments were conducted in parallel reaction monitoring mode with a resolution of 30,000 FWHM and an isolation window of *m*/*z* 1. Nitrogen was used as collision gas and generated using a CMC nitrogen generator (Eschborn, Germany). The MS was calibrated weekly with the calibration solution provided by Thermo Scientific.

### 3.5. Method Characterization

The LC-HRMS/MS method was characterized based on the WADA International Standard for Laboratories, with additional parameters added where deemed necessary [21]. The imprecision was determined by calculating the relative standard deviation of 10 samples at 0.2, 1, and 2 ng/mL, both inter- and intra-day. The limit of detection and identification was determined by analyzing 10 samples at different concentration levels and determining the concentration at which 95% of samples would result in a positive finding by employing a sigmoidal fit function. The detection criteria were the presence of two HRMS/MS ion transitions that were confirmed visually. Identification criteria were the presence of three HRMS/MS ion transitions meeting mandatory requirements outlined in the applicable WADA technical document TD 2021 IDCR [22]. The selectivity was determined by analyzing a set of 10 blank urine samples from both men and women with a range of pH values and SG and inspecting them for interfering signals. Recovery was determined by comparing samples in which the analyte and ISTD was added after the sample work-up to a set of samples in which the analyte was added at the beginning and the ISTD was added after the work-up. Matrix effects were determined by comparing the peak areas of ten spiked urine samples with one water sample that was spiked with the analyte. For the determination of extract stability, worked-up samples were stored for 7 days in the autosampler before re-analysis. Carryover was determined by measuring a prepared blank sample directly after a spiked sample at a high concentration. The robustness was determined by changing steps in the work-up procedure (samples were frozen after extraction and *t*BME was decanted the next day; the samples were dried in a vacuum centrifuge and reconstituted in 0.1% FA/ACN (80/20, *v*/*v*)) and comparing to samples after the standard work-up procedure. Additionally, the relative standard deviation of the retention times was calculated. For the linearity, calibration curves from 6 different days were aggregated.

### 3.6. Biotransformer

For initial metabolism simulation, the online biotransformer tool was used [19]. The software was operated in Phase I and Phase II mode, and three consecutive reaction steps were performed. SMILES was used as input format.

## 4. Conclusions

The interest in data regarding the excretion and metabolism of emerging doping substances has received increasing attention because the best-possible target analytes for adequate analytical retrospectivity need to be identified, and also, the problem of unintentional doping via contaminated dietary supplements necessitates further analytical data in support of case management. RAD140, a so-far unapproved SARM which is prohibited by WADA for its performance-enhancing effects, has led to multiple AAFs in recent years. In this study, a total of 30 controlled micro-dose administrations with healthy male volunteers were conducted, and the collected urine samples were analyzed by means of LC-HRMS/MS. Three novel metabolites were identified, as well as four metabolites that were previously only detected in equine samples. Using a newly developed and characterized method, the elimination profiles and detection times of RAD140 were generated, and the accumulation behavior after multi-dose application was described. For multiple metabolites, detection time ranges were determined for the different ingested doses of RAD140. Seven metabolite ratios were investigated for their predictive power regarding the time of intake of the SARM. From this data, tentative “threshold ratios” were postulated that assist in cases of AAFs involving RAD140 by estimating the time of ingestion. Additional elimination studies with higher doses of RAD140 may help in corroborating these threshold ratios and add to the existing data of the metabolism and elimination behavior of RAD140.

## Figures and Tables

**Figure 3:**
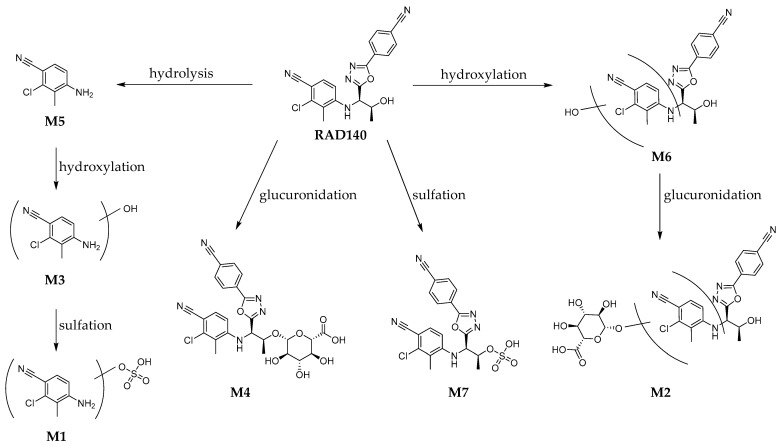
Proposed metabolic pathway of the detected metabolites of RAD140 in humans.

**Table 1:** List of discovered metabolites of RAD140 in human urine by means of LC-HRMS/MS.

Analyte	Transformation	Molecular Formular	RT [min]	Polarity	Precursor Ions *m*/*z*	Product Ions *m*/*z*	NCE [%]	Ref.
RAD140	-	C_18_H_11_ClN_5_O^−^	6.84	−	348.0658	321.0549 127.0302 175.0068	20	[11]
		C_20_H_17_ClN_5_O_2_^+^		+	394.1065	223.0633 205.0527 172.0505	30	[12]
M1	hydrolysis hydroxylation sulfation	C_8_H_6_ClN_2_O_4_S^−^	3.54	−	260.9742	181.0174 260.9742	25	[12]
M2a	glucuronidation hydroxylation	C_26_H_23_ClN_5_O_9_^−^	4.59	−	584.1190	193.0354 540.0928 364.0607	30	
M2b	glucuronidation hydroxylation		4.79	−		193.0354 390.0763 170.0360	30	
M3	hydrolysis hydroxylation	C_8_H_6_ClN_2_O^−^	4.98	−	181.0174	145.0407 118.0298	50	[12]
M4	glucuronidation	C_26_H_23_ClN_5_O_8_^−^	5.42	−	568.1241	193.0354 113.0244 374.0814	30	[12]
M5	hydrolysis	C_8_H_8_ClN_2_^+^	5.49	+	167.0371	167.0371 131.0604 104.0495	50	[12]
M6a	hydroxylation	C_18_H_11_ClN_5_O_2_^−^	5.76	−	364.0607	170.0360 193.0174 145.0407	30	[11] ^1^
M6b	hydroxylation		5.91	−		170.0360 193.0174 145.0407	30	[11] ^1^
M6c	hydroxylation		6.59	−		180.0096 193.0174 145.0407	30	[11] ^1^
M7	sulfation	C_20_H_15_ClN_5_O_5_S^−^	6.25	−	472.0488	348.0658 96.9601	30	
S-24	(ISTD ^2^)	C_18_H_13_O_3_N_2_F_4_^−^	7.45	−	381.0868	241.0594	30	[20]

^1^ Only two hydroxy metabolites were reported by Sobolevsky et al. ^2^ Internal standard.

**Table 2:** Method characterization results of the LC-HRMS/MS method for the detection of RAD140.

Intra-day imprecision	15.0%	at 2 ng/mL	*n* = 10
Intra-day imprecision	12.8%	at 1 ng/mL	*n* = 10
Intra-day imprecision	20.0%	at 0.2 ng/mL	*n* = 10
Inter-day imprecision	18.5%	at 2 ng/mL	*n* = 30
Inter-day imprecision	14.9%	at 1 ng/mL	*n* = 30
Inter-day imprecision	23.4%	at 0.2 ng/mL	*n* = 30
LOD	9	pg/mL	*n* = 10
Selectivity	yes		*n* = 10
LOI	120	pg/mL	*n* = 10
Recovery	20.6–70.2%	at 1 ng/mL	*n* = 10
Matrix effects	94.2–122.1%	at 1 ng/mL	*n* = 10
Extract stability (7 days, 4 °C)	55.9–93.4%	at 1 ng/mL	*n* = 10
Carryover	0.0%	at 8 ng/mL	*n* = 1
Robustness	12.4%		*n* = 10
STDev retention times	0.05%		*n* = 10
Linearity (R^2^)	0.9918–0.9990		*n* = 6

**Table 3:** Detection times of metabolites after micro-dose application of RAD140. The range is between the first sample and the last sample in which the analyte was detectable in any of the volunteers (*n* = 5).

Analyte	1·1 μg	5·1 μg ^1^	1·10 μg	5·10 μg ^1^	1·50 μg	5·50 μg ^1^
RAD140	2–212 h	0 ^2^–240 h	2–240 h	0 ^2^–501 h	2–696 h	0 ^2^–912 h
M1	-	-	28 h ^3^	0 ^2^–118 h	2–120 h	0 ^2^–168 h
M3	-	2–72 h	2–118 h	0 ^2^–185 h	2–333 h	0 ^2^–503 h
M5	-	-	-	0 ^2^–45 h	2–597 h	0 ^2^–190 h
M6a	-	-	35–72 h	0 ^2^–162 h	4–288 h	0 ^2^–406 h
M6b	10–32 h	0 ^2^–96 h	2–223 h	0 ^2^–282 h	2–504 h	0 ^2^–646 h
M6c	-	-	4–30 h	0 ^2^–115 h	2–261 h	0 ^2^–406 h

^1^ Values are calculated from the last ingestion of RAD140. ^2^ Analyte is detectable in sample before last ingestion due to multi-dose application. ^3^ Analyte was detected in a single sample.

**Table 4:** Tentative “threshold ratios” of different metabolite combinations. All measured abundance ratios of the 50 μg application studies were included (*n* = 5 + 5).

Presumed Time Point of Drug Intake	M6b/ M6c	M6a/ M6c	M6a/ M6b	M6b/ M3	M6a/ M3	M6a/ RAD140	M6b/ RAD140
<48 h	<12	<0.8	<0.05	<2.5	-	-	<0.15
<72 h	-	-	-	-	<0.2	<0.03	-
>96 h	>50	>8	-	-	-	-	-

## Data Availability

Data are contained within the article or Supplementary Material.

## Supplementary Materials

The following supporting information can be downloaded at: https://www.mdpi.com/article/10.3390/metabo12070666/s1, Figure S1. HRMS/MS spectrum and structure of RAD140 (negative), Figure S2. HRMS/MS spectrum and structure of RAD140 (positive), Figure S3. HRMS/MS spectrum and structure of M1, Figure S4. HRMS/MS spectrum and postulated structure of M2a, Figure S5. HRMS/MS spectrum and postulated structure of M2b, Figure S6. HRMS/MS spectrum and structure of M3, Figure S7. HRMS/MS spectrum and postulated structure of M4, Figure S8. HRMS/MS spectrum and structure of M5, Figure S9. HRMS/MS spectrum and structure of M6a, Figure S10. HRMS/MS spectrum and structure of M6b, Figure S11. HRMS/MS spectrum and structure of M6c, Figure S12. HRMS/MS spectrum and postulated structure of M7, Figure S13. Chromatograms of RDS (a) after hydrolysis after 1:10 dilution (b) after direct injection, Figure S14. Elimination profile of RAD140 after the intake of a single dose of 1 μg RAD140, Figure S15. Elimination profile of RAD140 after the intake of a single dose of 10 μg RAD140, Figure S16. Elimination profile of RAD140 after the intake of five doses of 1 μg RAD140 over 5 days, Figure S17. Elimination profile of RAD140 after the intake of five doses of 10 μg RAD140 over 5 days, Figure S18. Metabolite ratios M6a/M3 of single application of RAD140 (orange) and multi-dose application of RAD140 (blue), Figure S19. Metabolite ratios M6a/M6b of single application of RAD140 (orange) and multi-dose application of RAD140 (blue), Figure S20. Metabolite ratios M6a/M6c of single application of RAD140 (orange) and multi-dose application of RAD140 (blue), Figure S21. Metabolite ratios M6a/RAD140 of single application of RAD140 (orange) and multi-dose application of RAD140 (blue), Figure S22. Metabolite ratios M6b/M3 of single application of RAD140 (orange) and multi-dose application of RAD140 (blue), Figure S23. Metabolite ratios M6b/RAD140 of single application of RAD140 (orange) and multi-dose application of RAD140 (blue).
