# Supplementary material for: Human In Vivo Metabolism and Elimination Behavior of Micro-Dosed Selective Androgen Receptor Modulator RAD140 for Doping Control Purposes
Source: Metabolites. 2022 Jul 20;12(7):666. doi: 10.3390/metabo12070666 (PMC9325264; doi:10.3390/metabo12070666)
Supplement: Supplementary file 1 [file metabolites-12-00666-s001.zip › metabolites-1819443-supplementary.pdf]

metabolites

Human In Vivo Metabolism and Elimination Behavior of Micro-dosed Selective Androgen Receptor Modulator RAD140 for Doping Control Purposes

Felicitas Wagener [1], Luisa Euler[1], Christian Görgens[1], Sven Guddat[1] and Mario Thevis [1,2,*]

[1] Institute of Biochemistry/Center for Preventive Doping Research, German Sports University Cologne, Am Sportpark Müngersdorf 6, 50933 Cologne, Germany; f.wagener@biochem.dshs-koeln.de (F.W.); l.euler@biochem.dshs-koeln.de (L.E.); c.goergens@biochem.dshs-koeln.de (C.G.); s.guddat@biochem.dshs-koeln.de (S.G.)
[2] European Monitoring Center for Emerging Doping Agents (EuMoCEDA), 50933 Cologne, Germany

Supplementary Material

Figure S1. HRMS/MS spectrum and structure of RAD140 (negative), sample: RDS, urine sample was diluted 1:10 with water and prepared by hydrolysis and LLE. NCE 20%, isolation window m/z 1. 3
Figure S2. HRMS/MS spectrum and structure of RAD140 (positive), sample: RDS, urine sample was diluted 1:10 with water and prepared by hydrolysis and LLE. NCE 20%, isolation window *m/z* 1................. 3
Figure S3. HRMS/MS spectrum and structure of M1, sample: RDS, urine sample was injected directly. NCE 25%, isolation window *m/z* 1. 4
Figure S4. HRMS/MS spectrum and postulated structure of M2a, sample: RDS, urine sample was injected directly. NCE 15%, isolation window *m/z* 1. 4
Figure S5. HRMS/MS spectrum and postulated structure of M2b, sample: RDS, urine sample was injected directly. NCE 20%, isolation window *m/z* 1. 5
Figure S6. HRMS/MS spectrum and structure of M3, sample: RDS, urine sample was diluted 1:10 with water and prepared by hydrolysis and LLE. NCE 50%, isolation window *m/z* 1. 5
Figure S7. HRMS/MS spectrum and postulated structure of M4, sample: RDS, urine sample was injected directly. NCE 15%, isolation window m/z 1. 6
Figure S8. HRMS/MS spectrum and structure of M5, sample: RDS, urine sample was diluted 1:10 with water and prepared by hydrolysis and LLE. NCE 30%, isolation window *m/z* 1. 6
Figure S9. HRMS/MS spectrum and postulated structure of M6a, sample: RDS, urine sample was diluted 1:10 with water and prepared by hydrolysis and LLE. NCE 15%, isolation window *m/z* 1. 7
Figure S10. HRMS/MS spectrum and postulated structure of M6b, sample: RDS, urine sample was diluted 1:10 with water and prepared by hydrolysis and LLE. NCE 15%, isolation window *m/z* 1. 7
Figure S11. HRMS/MS spectrum and postulated structure of M6c, sample: RDS, urine sample was diluted 1:10 with water and prepared by hydrolysis and LLE. NCE 20%, isolation window *m/z* 1. 8
Figure S12. HRMS/MS spectrum and postulated structure of M7, sample: RDS, urine sample was injected directly. NCE 25%, isolation window *m/z* 1. 9

Figure S13. Chromatograms of RDS (a) after hydrolysis after 1:10 dilution (b) after direct injection. 10
Figure S14. Elimination profile of RAD140 after the intake of a single dose of 1 µg RAD140. The black line indicates the average values of the five volunteers, minimum and maximum values are shown as error bars. 10
Figure S15. Elimination profile of RAD140 after the intake of a single dose of 10 µg RAD140. The black line indicates the average values of the five volunteers, minimum and maximum values are shown as error bars. 11
Figure S16. Elimination profile of RAD140 after the intake of five doses of 1 µg RAD140 over 5 days. The black line indicates the average values of the five volunteers, minimum and maximum values are shown as error bars. 11
Figure S17. Elimination profile of RAD140 after the intake of five doses of 10 µg RAD140 over 5 days. The black line indicates the average values of the five volunteers, minimum and maximum values are shown as error bars. 12
Figure S18. Metabolite ratios M6a/M3 of single application of RAD140 (orange) and multi-dose application of RAD140 (blue). Arrows indicate the intake of 50 µg RAD140. 12
Figure S19. Metabolite ratios M6a/M6b of single application of RAD140 (orange) and multi-dose application of RAD140 (blue). Arrows indicate the intake of 50 µg RAD140. 13
Figure S20. Metabolite ratios M6a/M6c of single application of RAD140 (orange) and multi-dose application of RAD140 (blue). Arrows indicate the intake of 50 µg RAD140. 13
Figure S21. Metabolite ratios M6a/RAD140 of single application of RAD140 (orange) and multi-dose application of RAD140 (blue). Arrows indicate the intake of 50 µg RAD140. 14
Figure S22. Metabolite ratios M6b/M3 of single application of RAD140 (orange) and multi-dose application of RAD140 (blue). Arrows indicate the intake of 50 µg RAD140. 14
Figure S23. Metabolite ratios M6b/RAD140 of single application of RAD140 (orange) and multi-dose application of RAD140 (blue). Arrows indicate the intake of 50 µg RAD140. 15

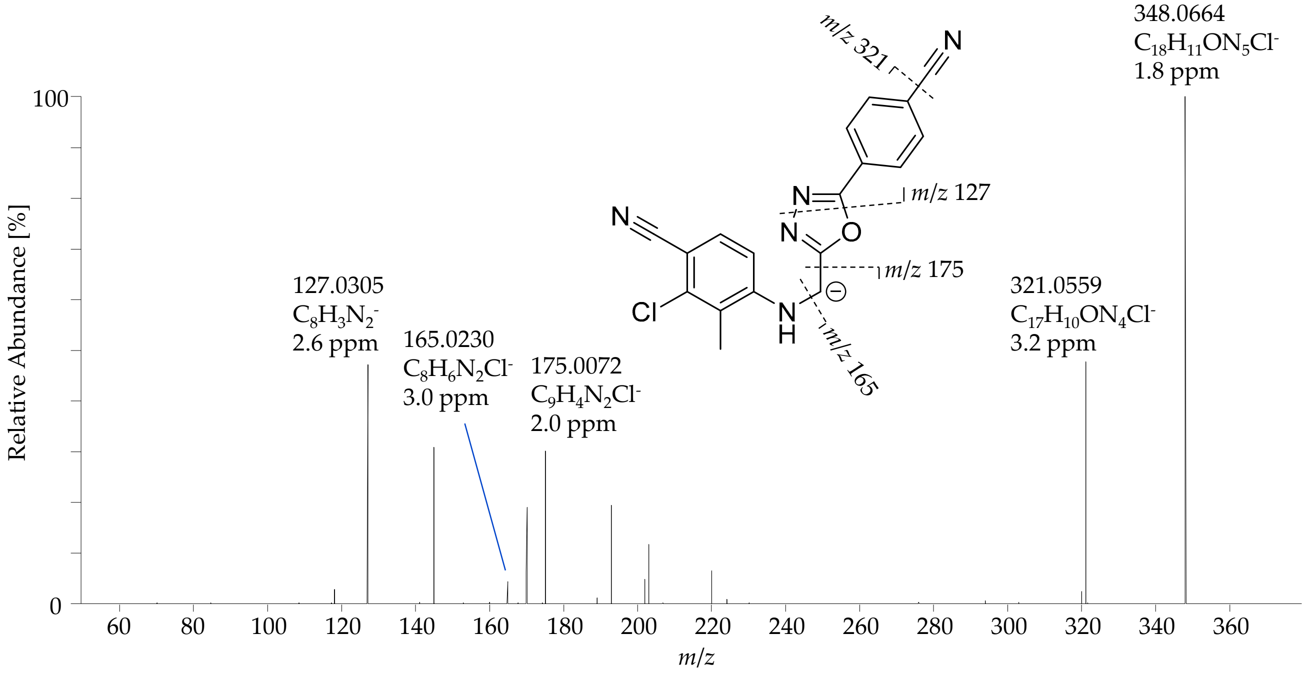

Figure S1. HRMS/MS spectrum and structure of RAD140 (negative), sample: RDS, urine sample was diluted 1:10 with water and prepared by hydrolysis and LLE. NCE 20%, isolation window m/z 1.

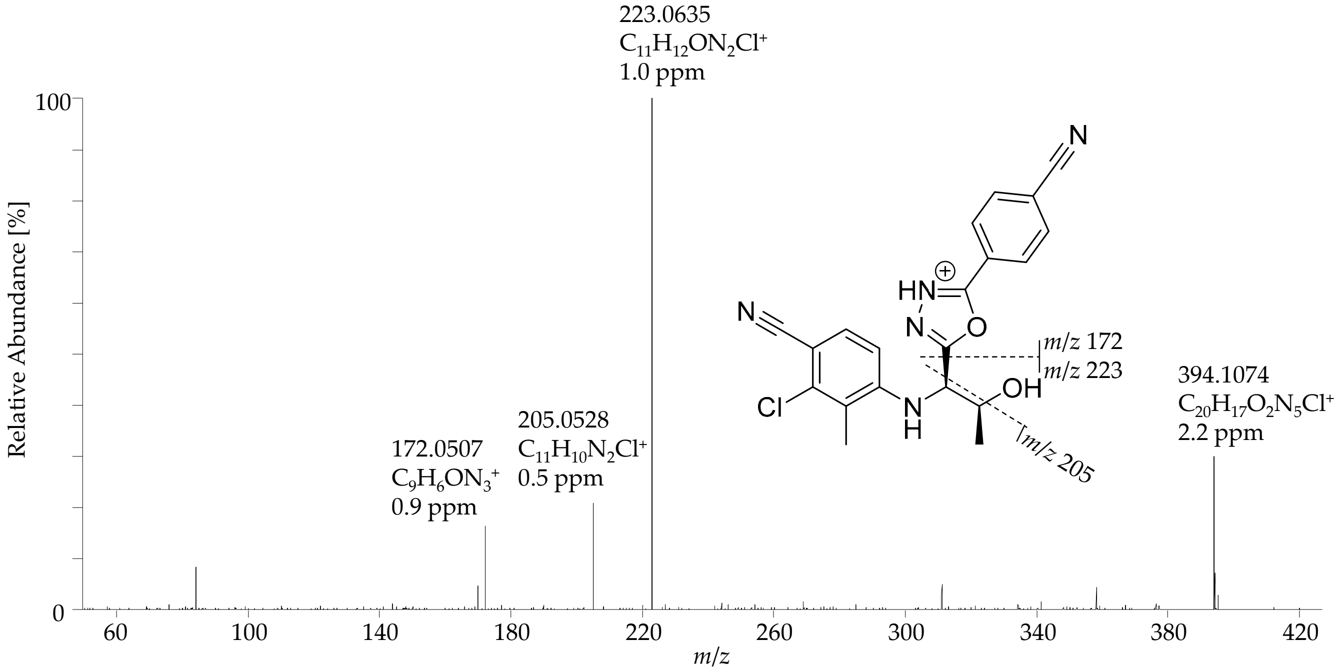

Figure S2. HRMS/MS spectrum and structure of RAD140 (positive), sample: RDS, urine sample was diluted 1:10 with water and prepared by hydrolysis and LLE. NCE 20%, isolation window *m*/*z* 1.

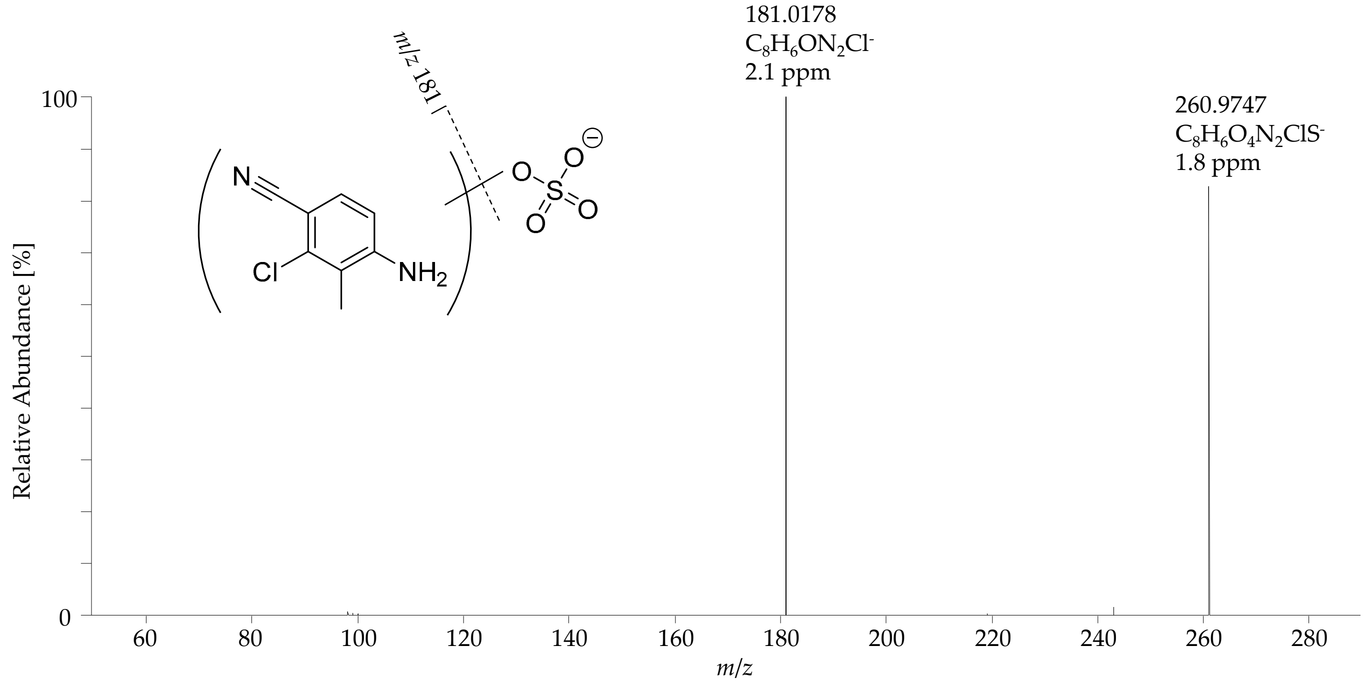

Figure S3. HRMS/MS spectrum and structure of M1, sample: RDS, urine sample was injected directly. NCE 25%, isolation window *m*/*z* 1.

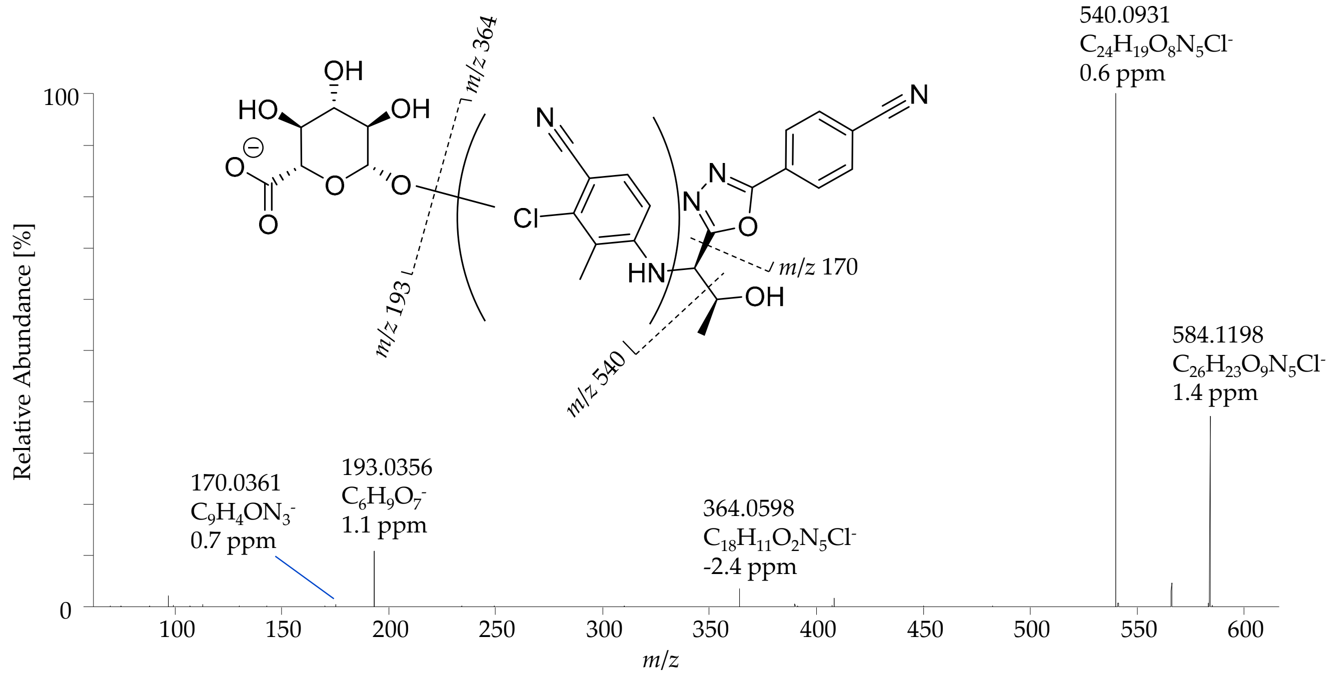

Figure S4. HRMS/MS spectrum and postulated structure of M2a, sample: RDS, urine sample was injected directly. NCE 15%, isolation window *m*/*z* 1.

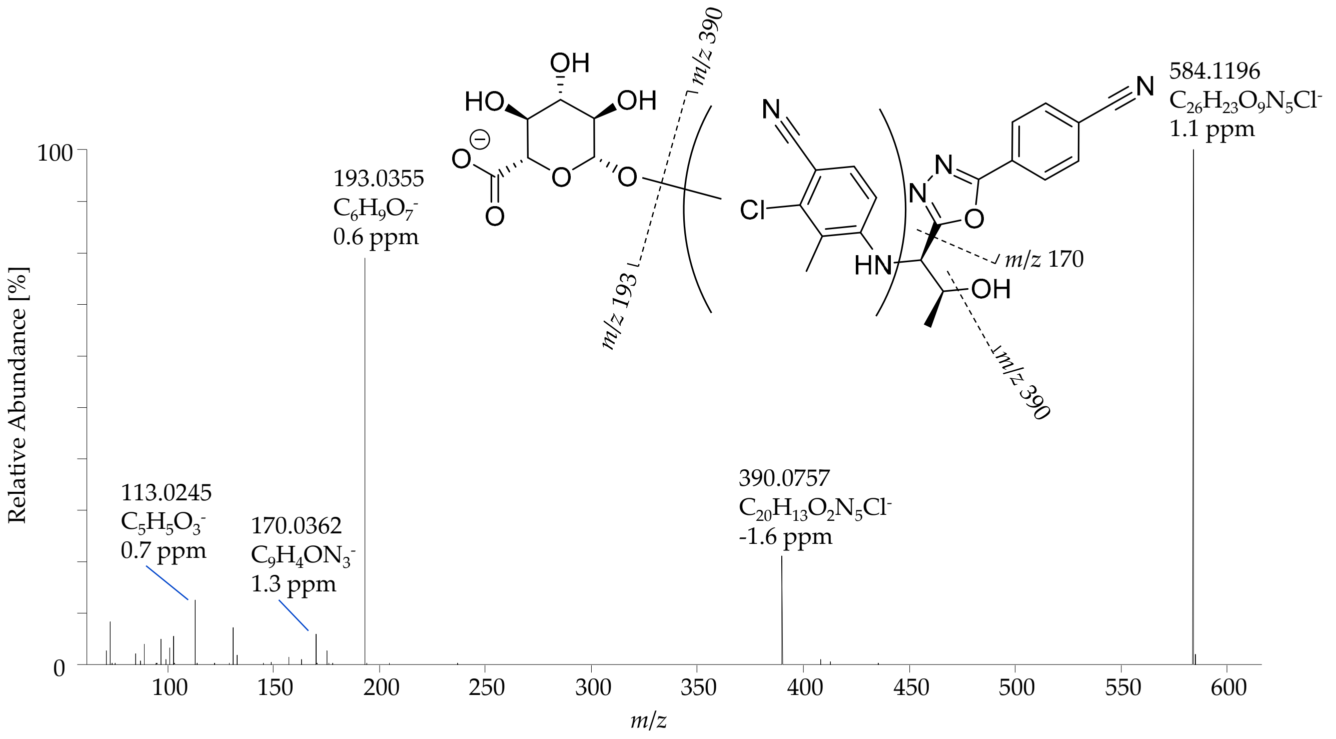

Figure S5. HRMS/MS spectrum and postulated structure of M2b, sample: RDS, urine sample was injected directly. NCE 20%, isolation window *m/z* 1.

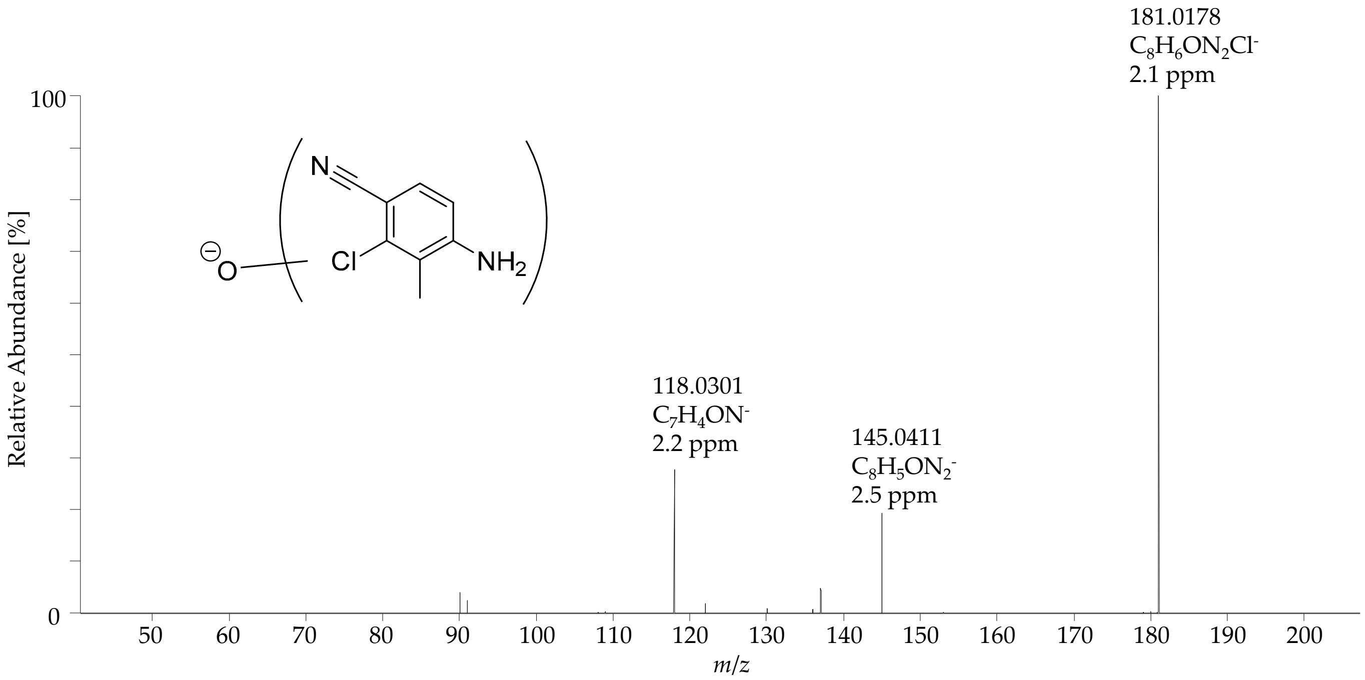

Figure S6. HRMS/MS spectrum and structure of M3, sample: RDS, urine sample was diluted 1:10 with water and prepared by hydrolysis and LLE. NCE 50%, isolation window *m/z* 1.

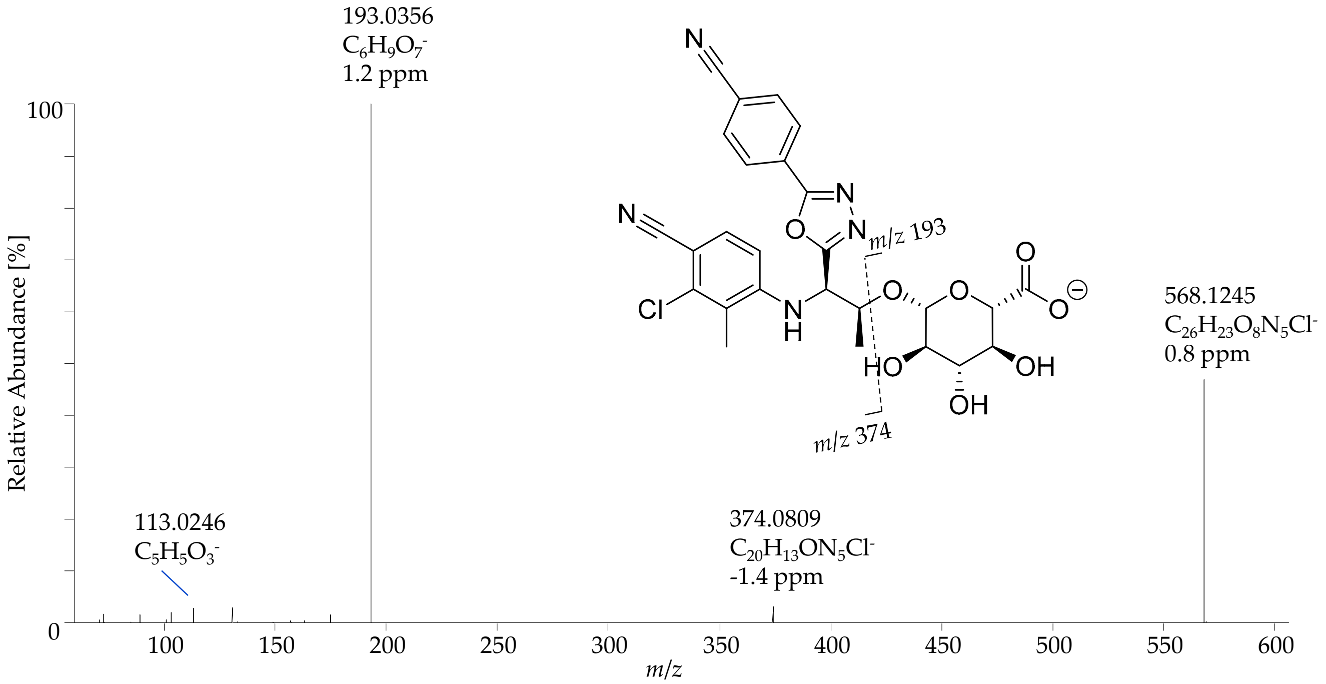

Figure S7. HRMS/MS spectrum and postulated structure of M4, sample: RDS, urine sample was injected directly. NCE 15%, isolation window m/z 1.

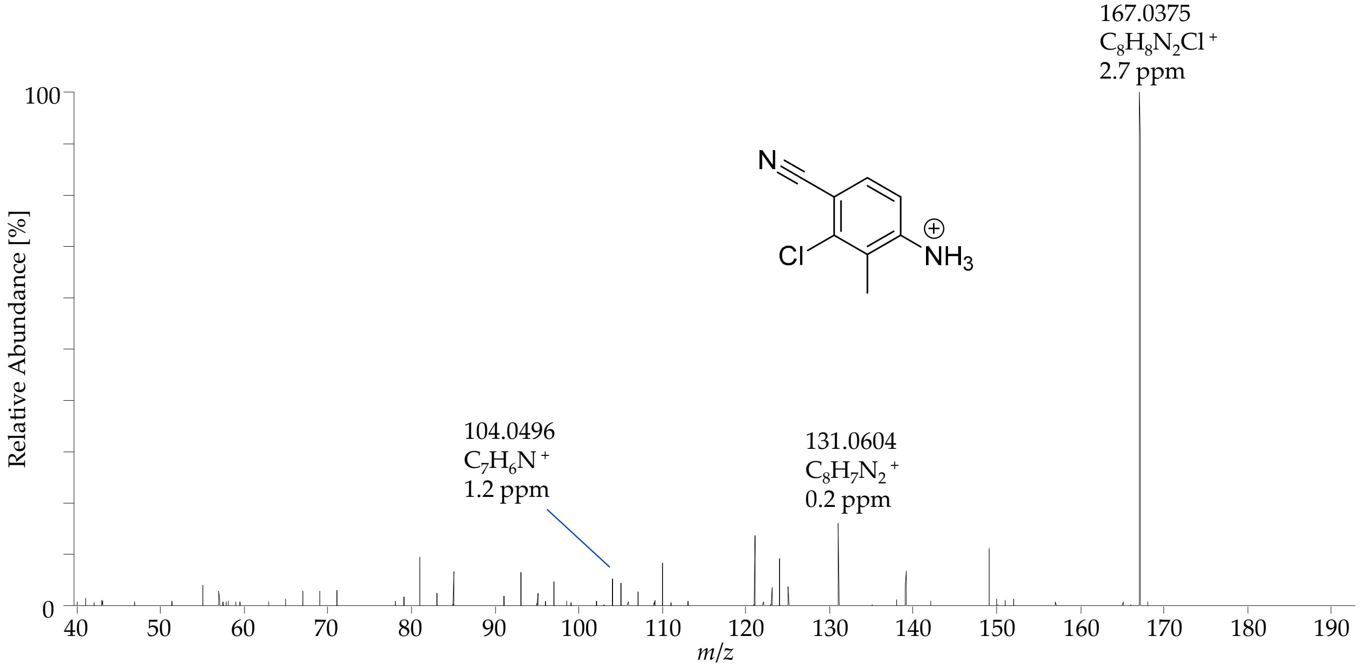

Figure S8. HRMS/MS spectrum and structure of M5, sample: RDS, urine sample was diluted 1:10 with water and prepared by hydrolysis and LLE. NCE 30%, isolation window *m*/*z* 1.

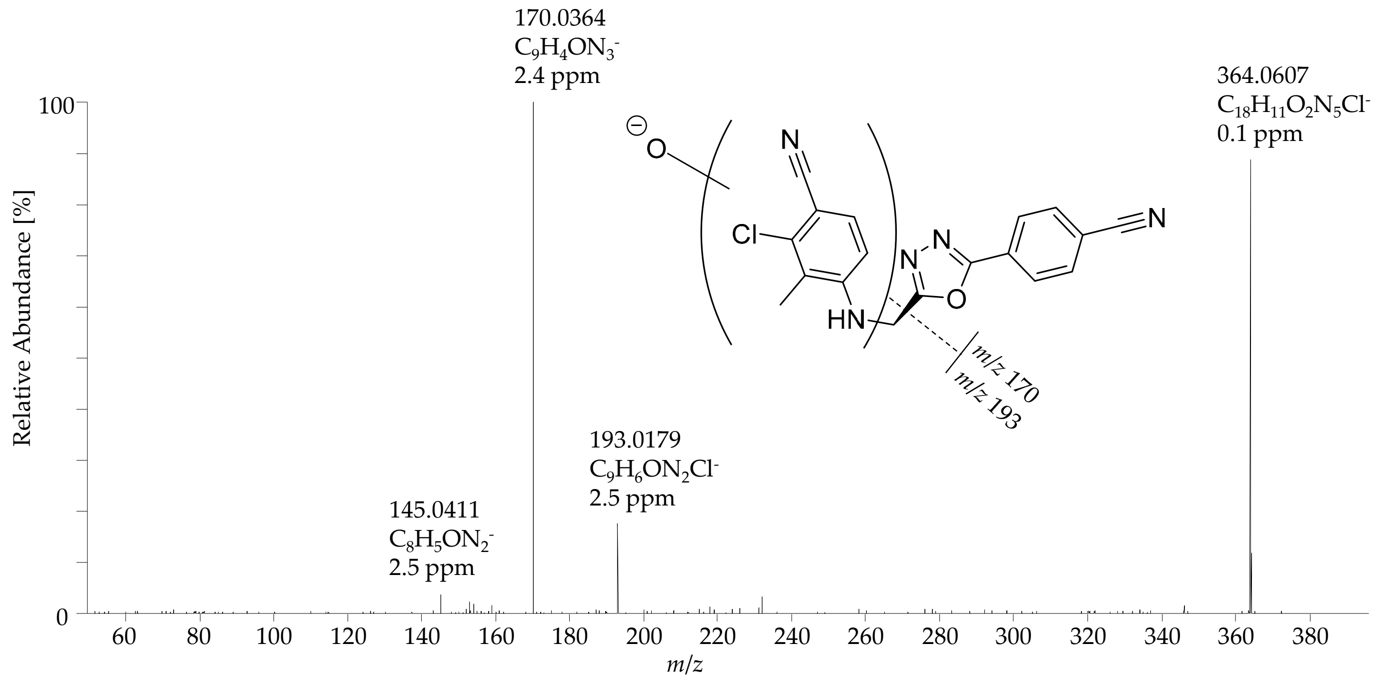

Figure S9. HRMS/MS spectrum and structure of M6a, sample: RDS, urine sample was diluted 1:10 with water and prepared by hydrolysis and LLE. NCE 15%, isolation window *m*/*z* 1.

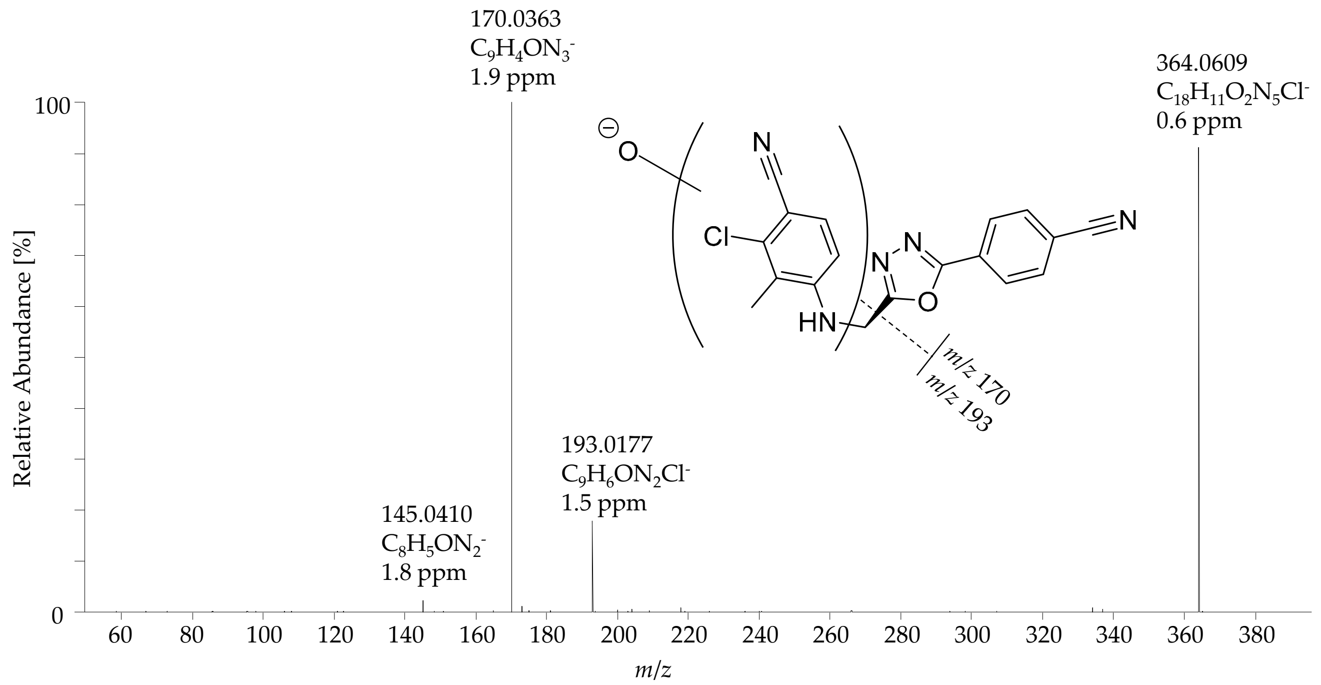

Figure S10. HRMS/MS spectrum and structure of M6b, sample: RDS, urine sample was diluted 1:10 with water and prepared by hydrolysis and LLE. NCE 15%, isolation window *m*/*z* 1.

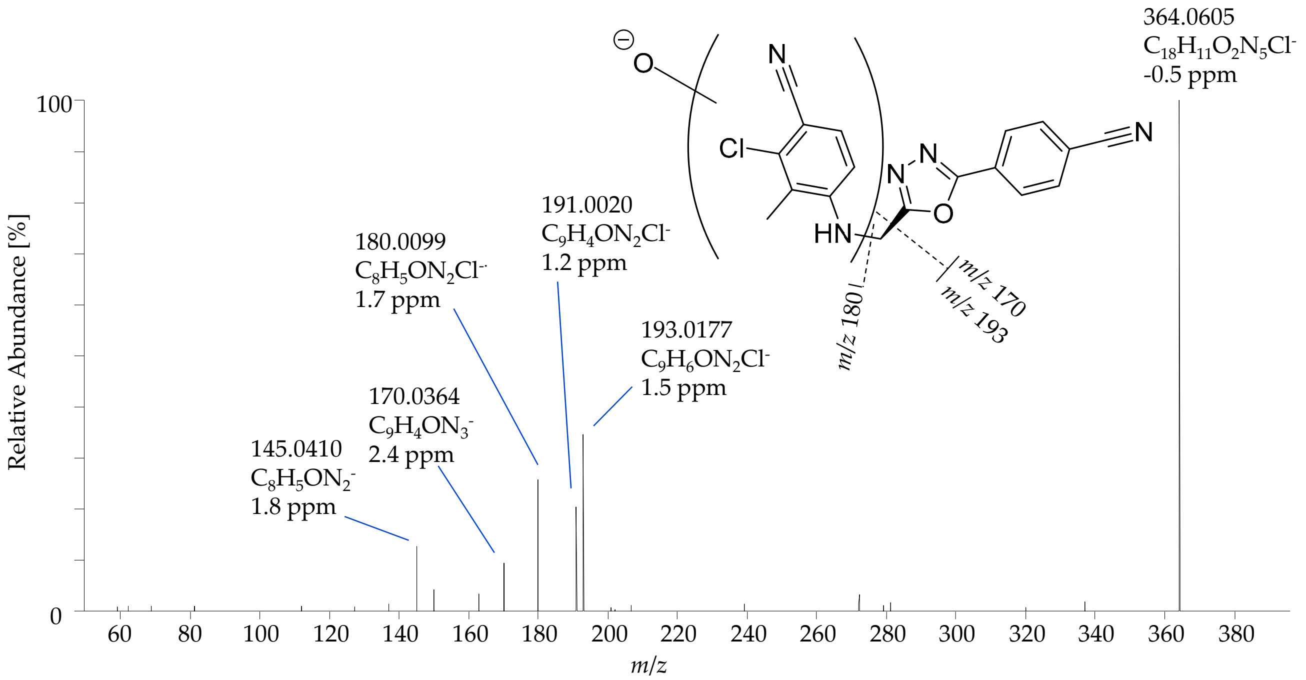

Figure S11. HRMS/MS spectrum and structure of M6c, sample: RDS, urine sample was diluted 1:10 with water and prepared by hydrolysis and LLE. NCE 20%, isolation window *m*/*z* 1.

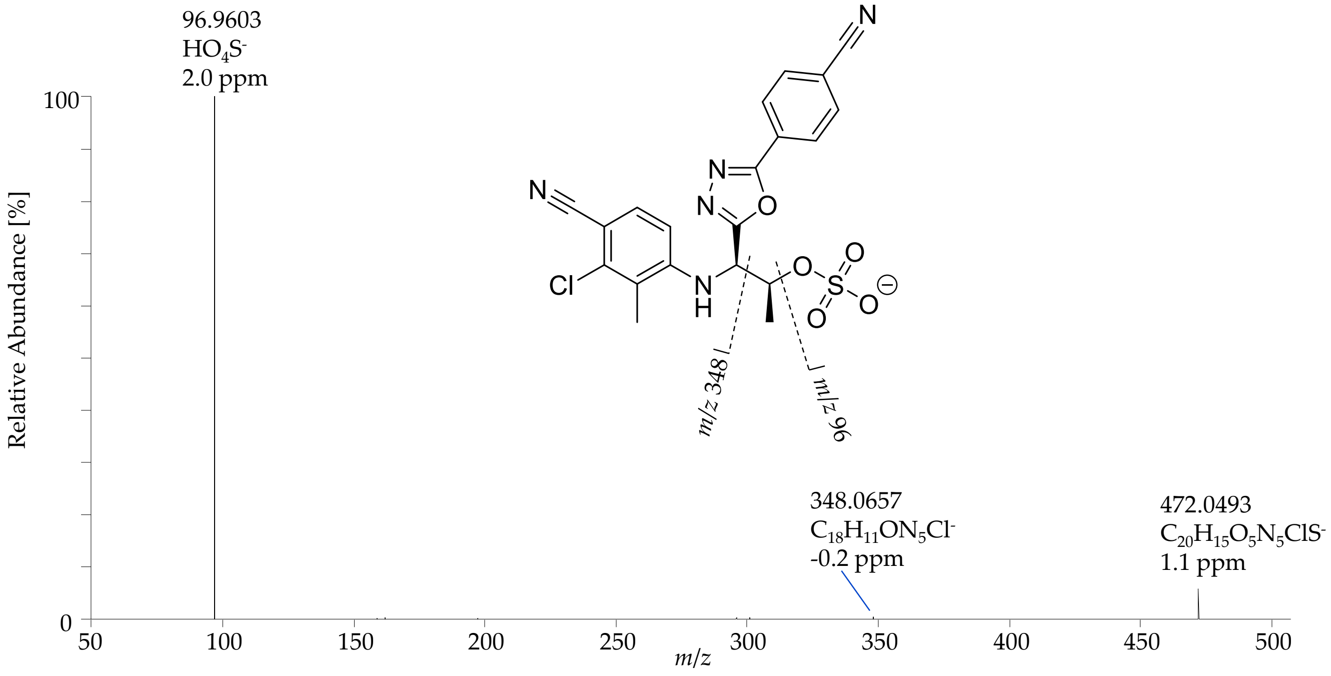

Figure S12. HRMS/MS spectrum and postulated structure of M7, sample: RDS, urine sample was injected directly. NCE 25%, isolation window *m*/*z* 1.

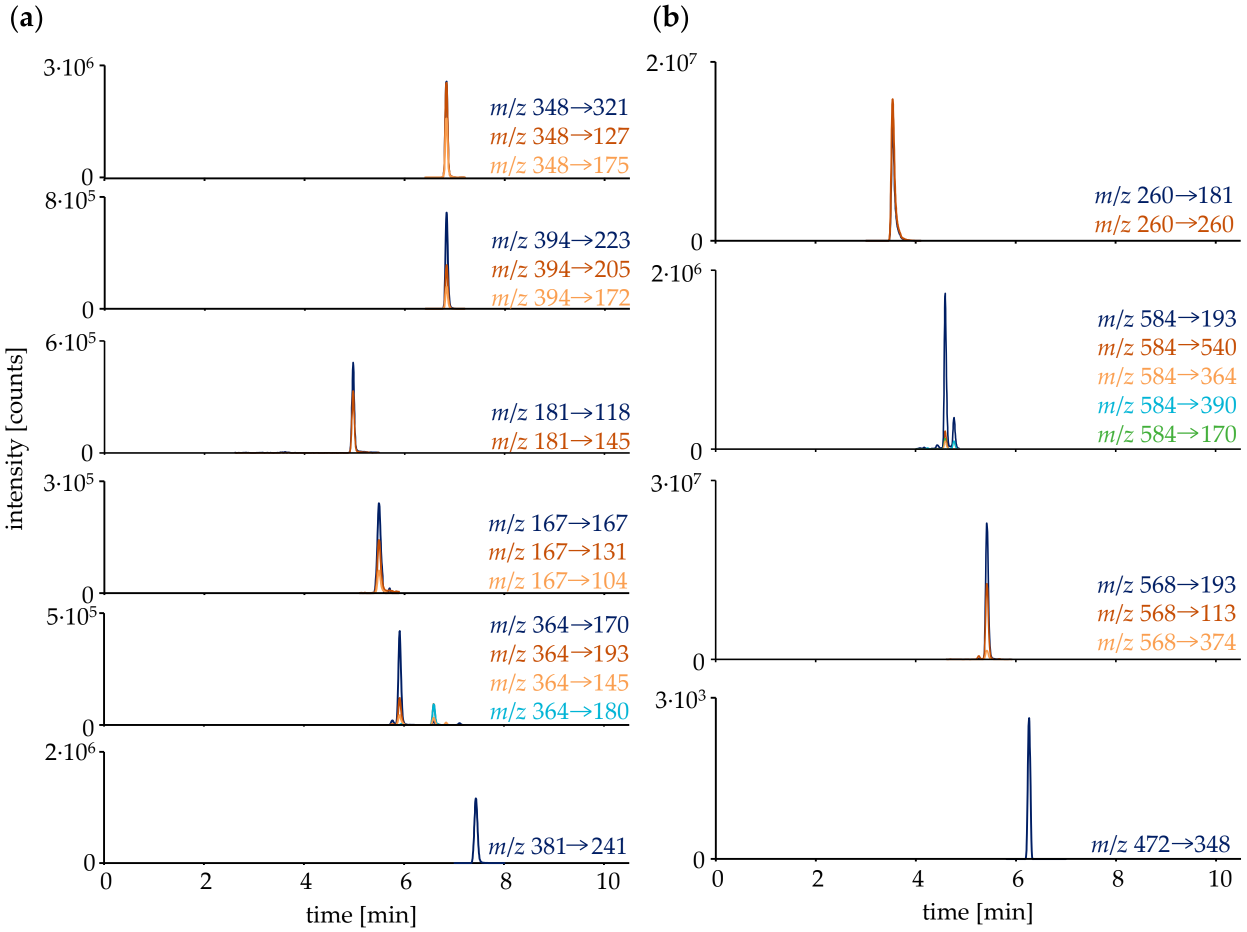

Figure S13. Chromatograms of RDS (a) after hydrolysis after 1:10 dilution (b) after direct injection.

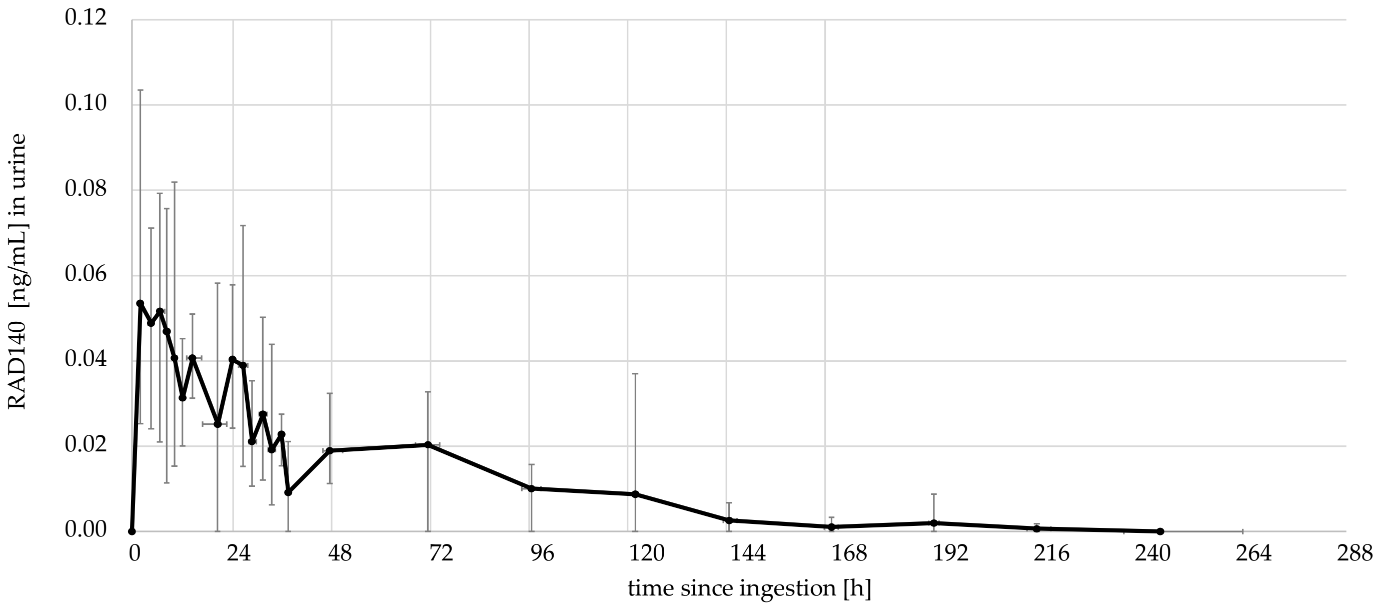

Figure S14. Elimination profile of RAD140 after the intake of a single dose of 1 µg RAD140. The black line indicates the average values of the five volunteers, minimum and maximum values are shown as error bars.

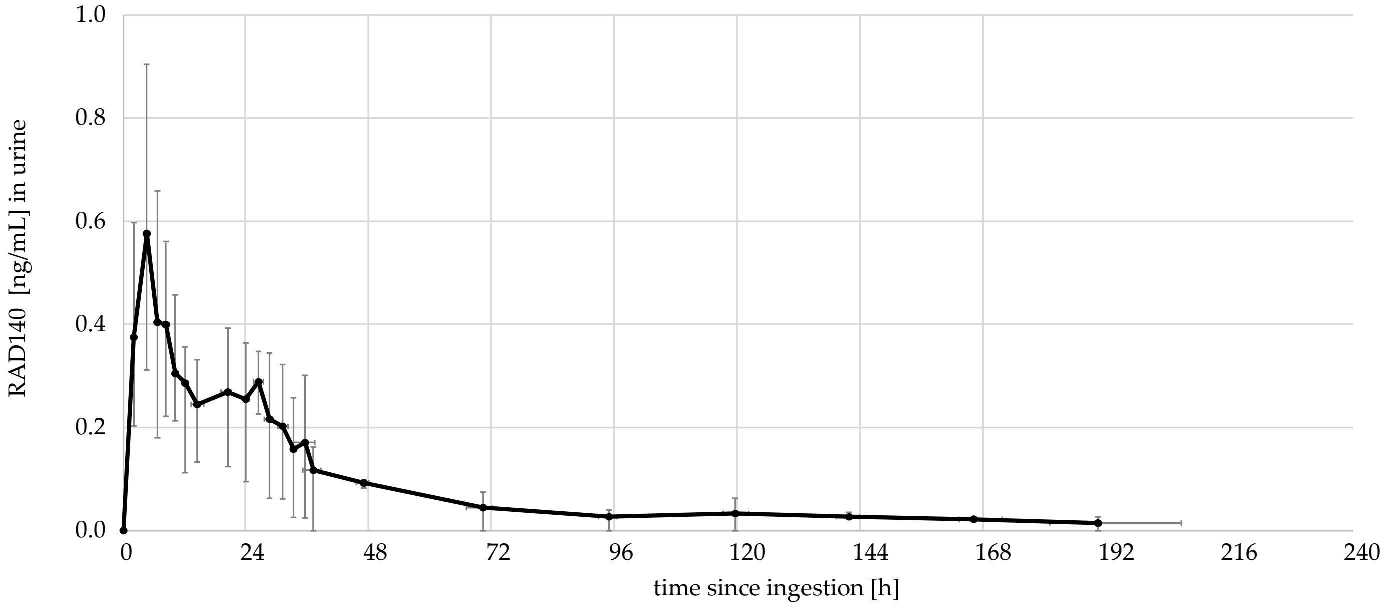

Figure S15. Elimination profile of RAD140 after the intake of a single dose of 10 µg RAD140. The black line indicates the average values of the five volunteers, minimum and maximum values are shown as error bars.

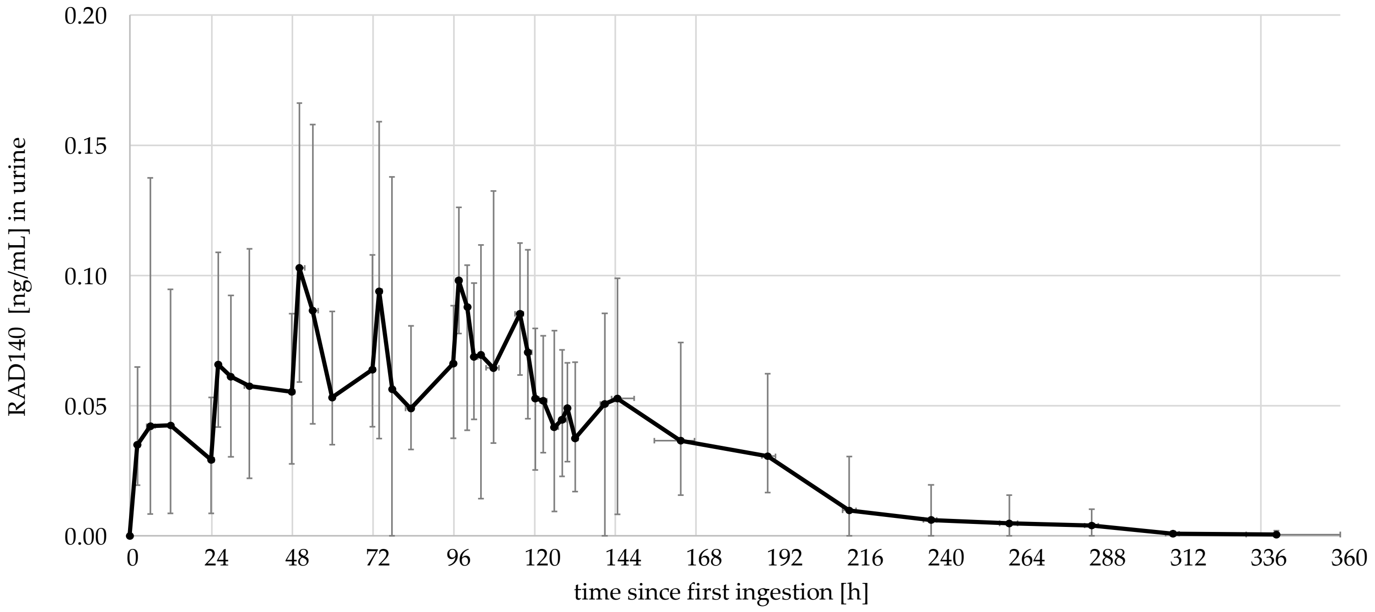

Figure S16. Elimination profile of RAD140 after the intake of five doses of 1 µg RAD140 over 5 days. The black line indicates the average values of the five volunteers, minimum and maximum values are shown as error bars.

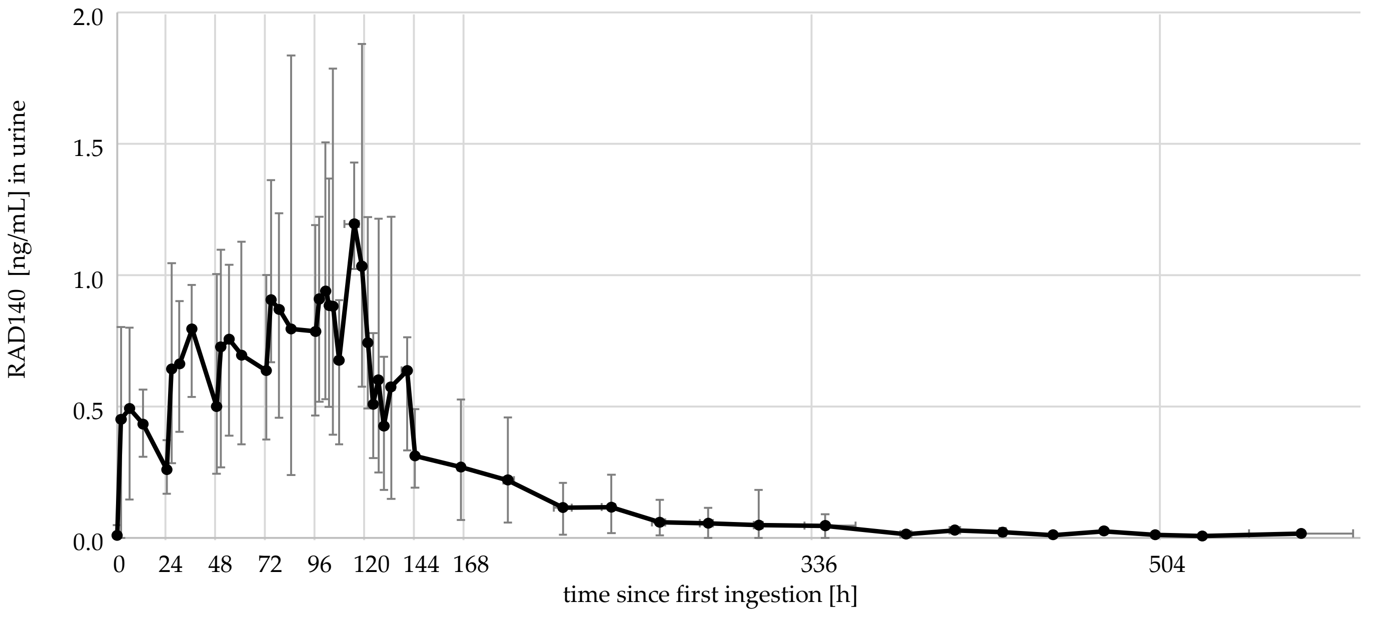

Figure S17. Elimination profile of RAD140 after the intake of five doses of 10 µg RAD140 over 5 days. The black line indicates the average values of the five volunteers, minimum and maximum values are shown as error bars.

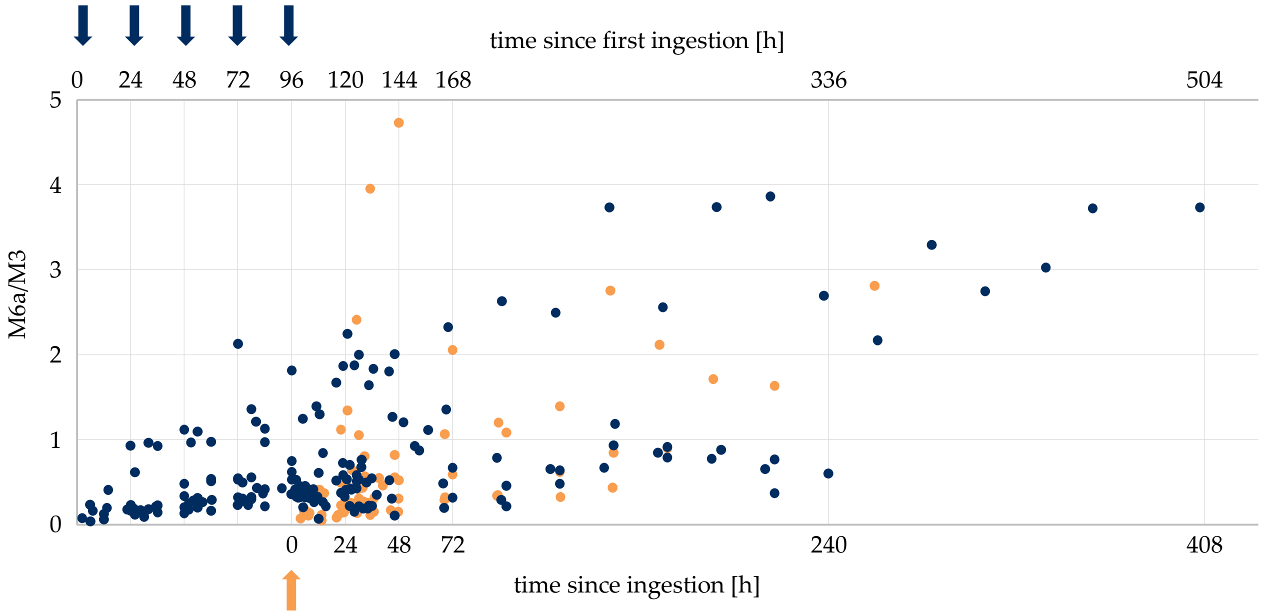

Figure S18. Metabolite ratios M6a/M3 of single application of RAD140 (orange) and multi-dose application of RAD140 (blue). Arrows indicate the intake of 50 µg RAD140.

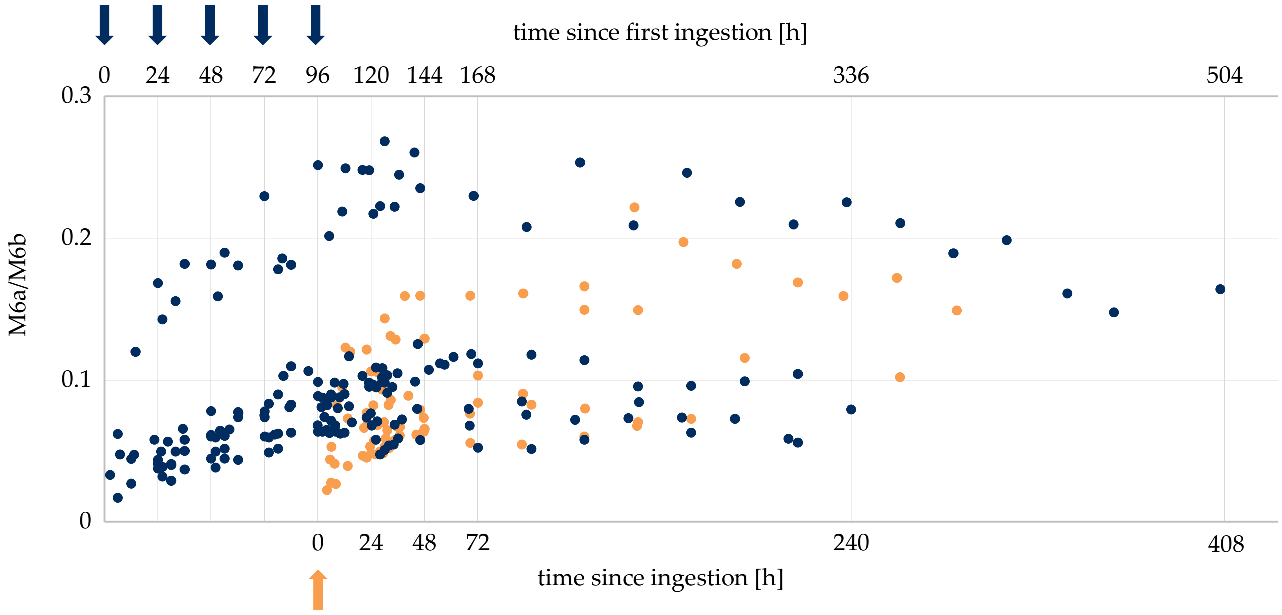

Figure S19. Metabolite ratios M6a/M6b of single application of RAD140 (orange) and multi-dose application of RAD140 (blue). Arrows indicate the intake of 50 µg RAD140.

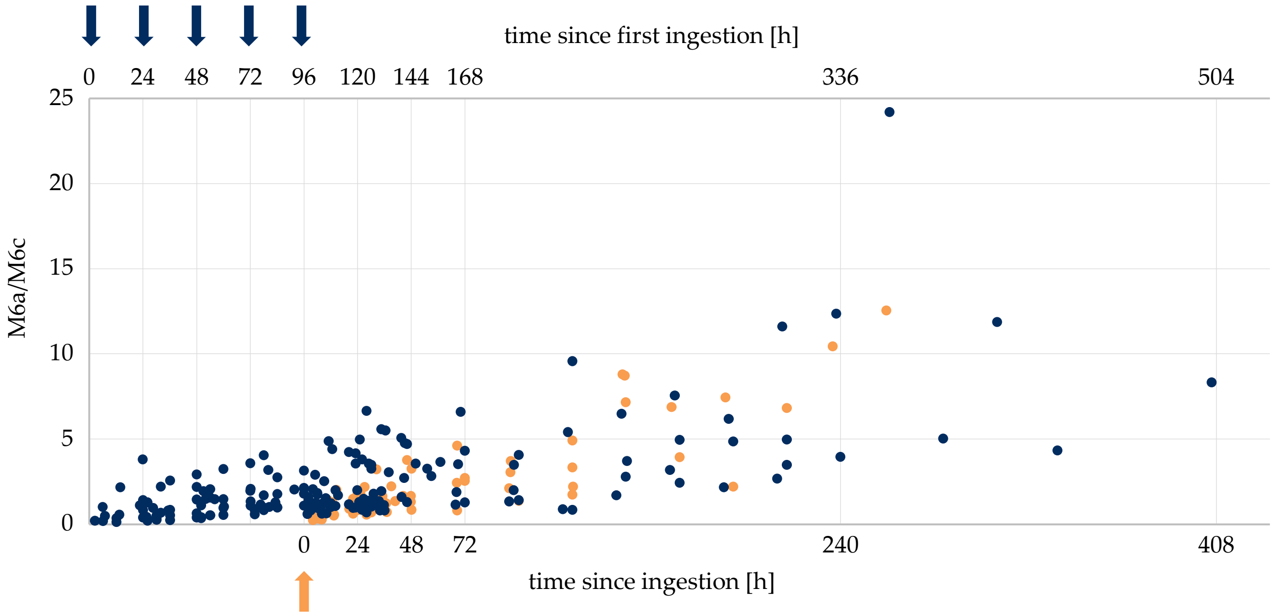

Figure S20. Metabolite ratios M6a/M6c of single application of RAD140 (orange) and multi-dose application of RAD140 (blue). Arrows indicate the intake of 50 µg RAD140.

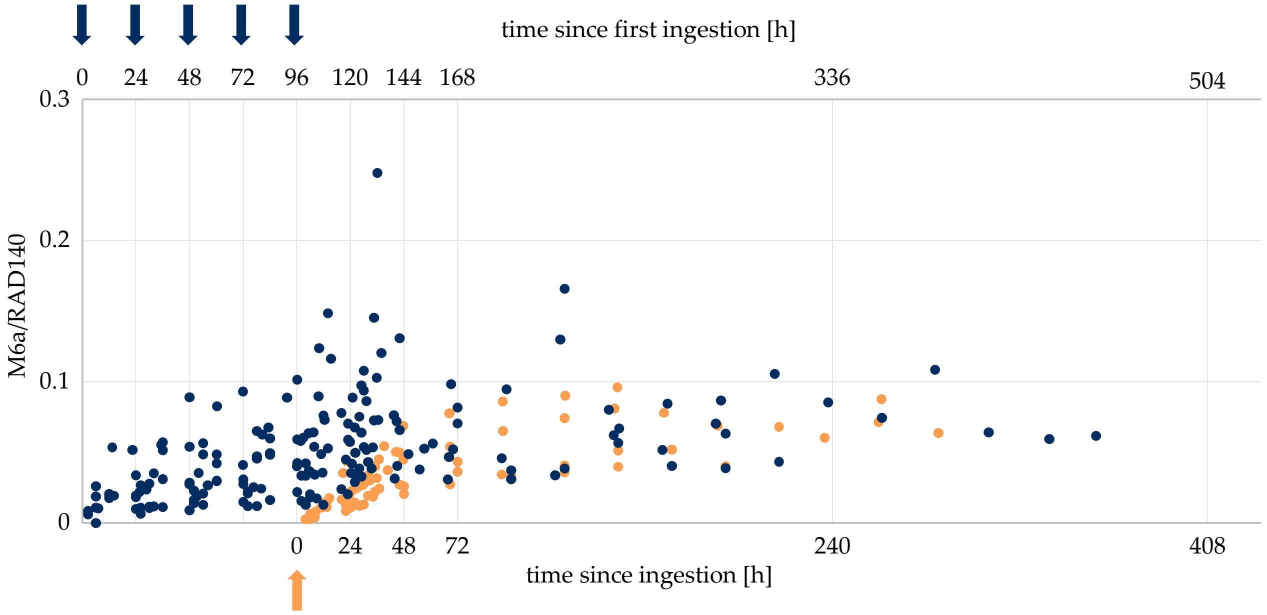

Figure S21. Metabolite ratios M6a/RAD140 of single application of RAD140 (orange) and multi-dose application of RAD140 (blue). Arrows indicate the intake of 50 µg RAD140.

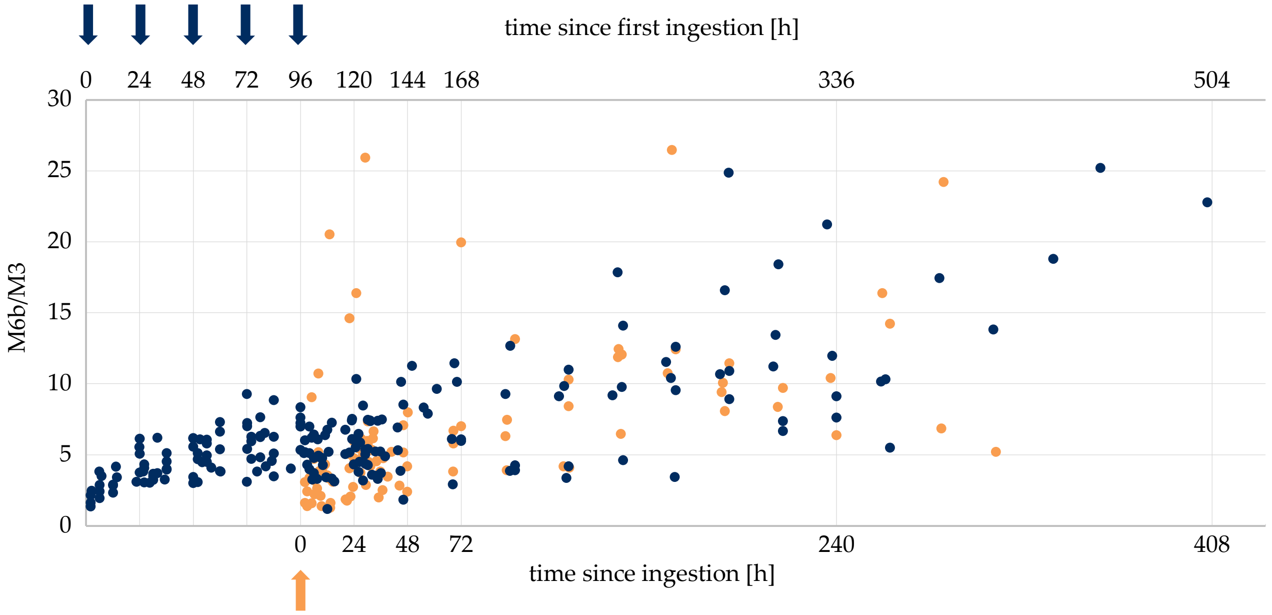

Figure S22. Metabolite ratios M6b/M3 of single application of RAD140 (orange) and multi-dose application of RAD140 (blue). Arrows indicate the intake of 50 µg RAD140.

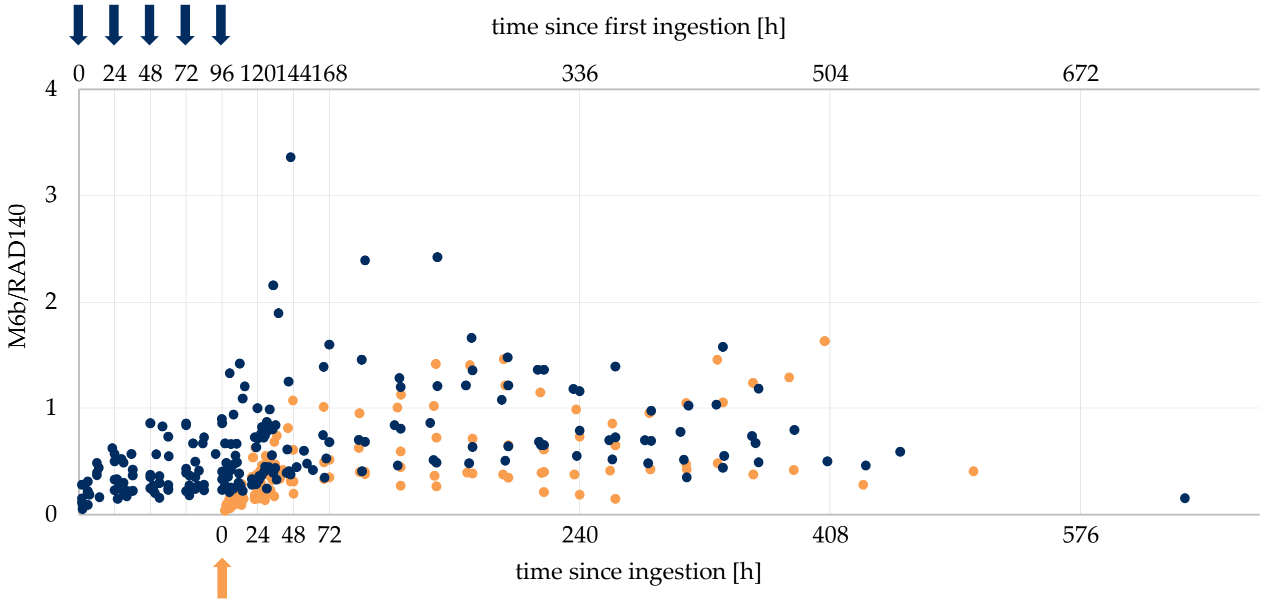

Figure S23. Metabolite ratios M6b/RAD140 of single application of RAD140 (orange) and multi-dose application of RAD140 (blue). Arrows indicate the intake of 50 µg RAD140.
